# Brainwaves under medication: revealing class-specific neural signatures of psychotropic medication from 24,000 EEGs

**DOI:** 10.1016/j.ebiom.2026.106375

**Published:** 2026-07-09

**Authors:** Magdalena Szponar, Patrycja Dzianok, Bartłomiej Gmaj, Wojciech Jernajczyk, Jan Kamiński

**Affiliations:** a Department of Neurosurgery, SUNY Upstate Medical University, Syracuse, NY, USA; b Laboratory of Neurophysiology of Mind, Nencki Institute of Experimental Biology, Warsaw, Poland; c International Institute of Molecular and Cell Biology in Warsaw, Warsaw, Poland; d Department of Psychiatry, Medical University of Warsaw, Warsaw, Poland; e Department of Clinical Neurophysiology, Institute of Psychiatry and Neurology, Warsaw, Poland

**Keywords:** Psychotropic drugs, Psychiatry, Pharmacotherapy, Mental disorders, Electroencephalography (EEG), PharmacoEEG

## Abstract

**Background:**

Psychotropic medications remain foundational in psychiatric care, yet the neurophysiological mechanisms through which they exert therapeutic and adverse effects are still poorly characterised, limiting the field's ability to optimise treatment selection and monitoring. Electroencephalography (EEG) offers a non-invasive, real-time window into brain function that could support more precise, mechanism-informed prescribing; however, progress has been constrained by the absence of sufficiently large and systematically analysed pharmaco-EEG datasets.

**Methods:**

In this cross-sectional observational study, we analysed over 24,000 clinical EEG recordings (∼6000 h of data) obtained across a wide range of psychiatric diagnoses and medication regimens. We compared more than 75,000 spectral, connectivity, and nonlinear EEG features across major drug classes, including benzodiazepines, SSRIs, antipsychotics, and anticonvulsants.

**Findings:**

Dimensionality-reduced analyses revealed robust, class-specific neurophysiological signatures that can be linked to psychotropic drugs' mechanisms of action: benzodiazepines increased beta and decreased theta–alpha power; SSRIs enhanced gamma-band coherence; and antipsychotics and anticonvulsants produced marked slow-wave amplification and reductions in signal complexity. All results are made publicly accessible through an interactive resource (BrainwavesRX), enabling clinicians and researchers to explore medication-specific EEG effects at multiple levels of granularity.

**Interpretation:**

By establishing a population-level reference atlas of psychotropic medication effects on human neural dynamics, this study provides an important foundation for future studies leveraging EEG to predict treatment response, detect insufficient or excessive pharmacological effects, and ultimately advance the development of individualised, data-driven psychiatric care.

**Funding:**

The publication was prepared as part of Foundation of Polish Science's Proof of Concept (FENG.02.01-IP.05-0010/24) and BRAINCITY IRAP (FENG.02.07-IP.05-0179/23) projects.

**Research in context**

**Evidence before this study**

Human studies examining the effects of psychotropic medications on EEG measures in clinical or healthy populations, found in PubMed, Scopus, and Web of Science (last accessed: September 2025, search terms: “pharmaco-EEG”, “quantitative EEG”, “resting-state EEG”, “psychotropic drugs”, “antidepressants”, “benzodiazepines”, “antipsychotics”, “antiepileptic drugs”, “spectral power”, “functional connectivity”, and “EEG complexity”) were examined.

Previous studies consistently show that psychotropic medications modulate EEG activity in frequency-specific patterns. Antidepressants are associated with reductions in alpha and increases in beta activity; benzodiazepines with increased beta power; antipsychotics and antiepileptic drugs with EEG slowing, reflected in increased delta and theta activity. However, most studies are small, focus primarily on spectral power, and examine single drug classes. Evidence for other EEG domains, including functional connectivity and nonlinear dynamics, is limited. Some medication classes—notably SARI and NaSSA antidepressants—remain insufficiently characterised, particularly in wakeful EEG. Overall, the evidence base is heterogeneous and at moderate risk of bias due to confounding by diagnosis, demographic factors, and small sample sizes. No large-scale study has systematically examined multiple EEG features across a broad range of psychotropic medications in a single dataset.

**Added value of this study**

This study provides a systematic analysis of resting-state EEG correlates of major psychotropic drug classes in a large clinical cohort of more than 20,000 patients. By jointly analysing spectral power, functional connectivity, and nonlinear dynamics, it extends beyond the single-feature focus of previous studies. The design accounts for demographic and diagnostic differences, enabling clearer attribution of EEG patterns to medication effects. The scale and scope of this dataset allow consistent cross-class comparisons and identification of robust electrophysiological signatures. The public release of results through an interactive interface provides a resource for reproducibility, hypothesis generation, and detailed exploration of drug–EEG associations.

**Implications of all the available evidence**

Available evidence, together with our findings, indicates that psychotropic medications are associated with distinct and partly class-specific EEG signatures that reflect their neuropharmacological mechanisms. These results support the need to account for medication effects when interpreting EEG biomarkers in psychiatric research. The comprehensive mapping presented here may serve as a reference for distinguishing medication-related changes from disease-related alterations and for guiding biomarker development. Future studies should prioritise longitudinal designs and mechanistic approaches to establish causal links between EEG changes, treatment response, and clinical outcomes.

## Introduction

Psychotropic medications are widely prescribed to manage a range of mental health disorders, including depression, schizophrenia, bipolar disorder, and anxiety disorders. They are also used as adjunctive treatments in addictions and personality disorders. While their therapeutic effects are well documented, the neurophysiological correlates of these treatments remain incompletely characterised. Electroencephalography (EEG) provides a non-invasive, high-temporal-resolution window into neural activity,^1^ making it a valuable tool for assessing how pharmacological interventions modulate brain bioelectrical activity. Pharmaco-EEG studies using clinical EEG and quantitative EEG (qEEG) technologies have existed for more than four decades.^2^ Numerous studies have demonstrated that psychotropic medications can modulate EEG patterns, including changes in spectral power, coherence, and event-related potentials, which may correspond to therapeutic effects or side effects.^3^, ^4^, ^5^ Investigating these alterations can improve our understanding of how psychotropic medication use is associated with brain bioelectrical activity in clinical populations. Moreover, medication-related EEG changes have been investigated as potential candidate biomarkers of treatment response.^6^, ^7^, ^8^ Understanding the influence of medicines on the EEG signal may help to disentangle the effects of medication and disorder in such studies. However, to interpret these EEG alterations in a meaningful way requires considering the pharmacological profiles of the medications with which they are associated. Different classes of psychotropic drugs act on distinct neurochemical systems, and these mechanisms of action could be reflected in characteristic patterns of EEG modulation. Therefore, before examining specific findings, it is useful to outline how major categories of psychotropic medications affect brain electrical activity.

**Fig. 1:**
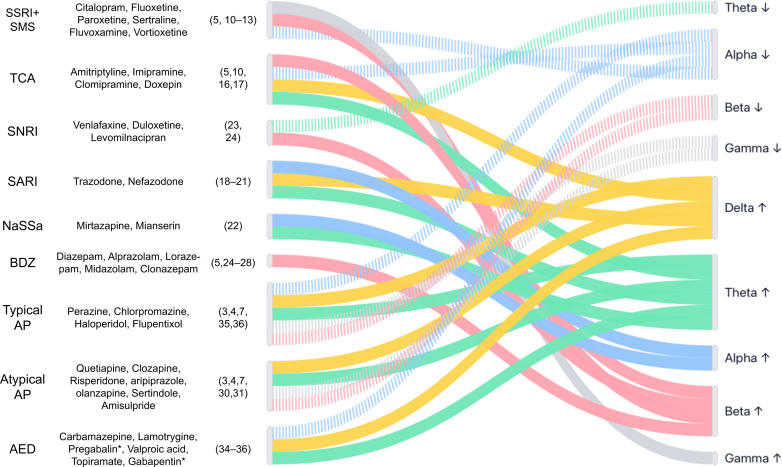
Graphic representation of the EEG effects described in the current literature, for the main substance groups, together with substance examples. Filled coloured lines represent an increase of power in a given frequency, while semi-transparent lines represent a decrease. ∗ Pregabalin and Gabapentin act by blocking calcium channels, unlike other anticonvulsants, which block sodium channels.

Most **antidepressants** influence serotonergic, noradrenergic, and dopaminergic transmission by blocking reuptake transporters or specific receptors.^9^ **Selective Serotonin Reuptake Inhibitors (SSRI)** typically decrease alpha and increase beta power,^5^^,^^10^, ^11^, ^12^, ^13^ with some agents (e.g., citalopram, vortioxetine) also enhancing gamma activity.^5^^,^^11^, ^12^, ^13^ **Serotonin-Norepinephrine Reuptake Inhibitors (SNRI)** show an increase in beta power^14^ and a decrease in theta-band power in the midline and right-frontal areas^15^ (see Fig. 1 for summary). Sedating **Tricyclic antidepressants (TCA)**, such as amitriptyline and imipramine, increase delta, theta, and fast-beta power while reducing alpha and total power,^5^^,^^10^^,^^16^^,^^17^ often slowing the alpha rhythm.^4^ While **Serotonin Receptor Antagonists with Serotonin Reuptake Inhibition (SARI)** produce well-documented increases in delta and theta power during sleep,^18^, ^19^, ^20^, ^21^ their effects on wakeful EEG remain poorly characterised. Likewise, wakeful EEG changes induced by **Noradrenergic and Specific Serotonergic Antidepressants (NaSSA)** are under-investigated, though limited evidence suggests that mianserin enhances theta and alpha activity.^22^

**Benzodiazepines (BDZ)** are anxiolytic drugs that target GABA_A_ receptors, prescribed predominantly for anxiety and mood disorders.^23^ Their most often reported effect on brain waves is an increase in beta power.^5^^,^^24^, ^25^, ^26^, ^27^, ^28^ Some studies also report a decrease in alpha power^25^^,^^28^ and an increase in fractal dimension^25^ after administration of BDZs.

**Antipsychotics** (AP, also called neuroleptics) antagonise dopaminergic transmission, thereby reducing positive psychotic symptoms such as hallucinations and delusions.^3^ They can be divided into typical (first-generation) or atypical (second-generation). Typical antipsychotics act almost exclusively on dopaminergic pathways, whereas atypical APs also modulate serotonin receptors.^29^ Both types of antipsychotics induce slowing of EEG rhythm, increasing delta and theta power,^3^^,^^4^^,^^7^^,^^30^^,^^31^ and decreasing fast beta power.^3^^,^^4^ Typical APs can also slow down the alpha rhythm.^4^^,^^30^ Some studies also show alterations in functional connectivity after antipsychotic treatment.^32^

**Anticonvulsants**, also known as antiepileptic drugs (AED), reduce seizure frequency in epilepsy by suppressing neuronal excitability or enhancing inhibitory neurotransmission,^33^ usually by blocking sodium or calcium channels. Their most typical effects on EEG are the overall attenuation of power and slowing down (usually increased power of delta and theta frequencies, decreased alpha power and slowing down alpha frequency peak).^34^, ^35^, ^36^

Fig. 1 presents a graphic summary of the known psychotropic drug effects on resting-state EEG. The majority of pharmaco-EEG studies have focused on spectral power changes, leaving other EEG dimensions–such as functional connectivity and signal complexity–often unexplored. Moreover, the wakeful EEG correlates of certain antidepressant classes (particularly SARI and NaSSA) remain insufficiently investigated.

In this study, we aim to address these gaps by systematically characterising the effects of major psychotropic drug classes on resting-state EEG in patients with neuropsychiatric disorders, using a large clinical dataset. We balance the groups based on demographic factors and psychiatric diagnosis, to increase confidence that the observed effects are related to medication status. By simultaneously investigating multiple EEG features — including connectivity, spectral power, and nonlinear dynamics — across a wide range of psychotropic medications, this work provides an integrative assessment of medication-related EEG differences that extends beyond the narrower focus of many previous studies. The dataset includes over 20,000 patients, providing substantial statistical power and supporting the robustness and generalisability of the findings. Together, this scale and multidimensional approach offer a comprehensive view of how different classes of psychotropic drugs correlate with resting-state brain activity. We make our results publicly available as an interactive interface, where anyone can display the full results, including graphs, test statistics, and the effect size measures for any of the multiple investigated features and drugs. Characterising associations between medication classes and electrophysiological measures may help organise future work linking pharmacology, brain activity, and clinical phenomena. Previous studies have reported associations between some medication-related EEG changes and symptom measures. For instance, drug-induced alterations in theta power have been associated with reductions in psychotic symptoms,^37^^,^^38^ while increases in beta power following benzodiazepine treatment correlate with decreased anxiety.^39^ A more systematic description of medication-associated EEG signatures may provide a reference framework for future studies aimed at distinguishing therapeutic drug electrophysiological changes–such as normalisation of gamma phase synchronisation in patients with schizophrenia after antipsychotic treatment, as Alegre et al.^40^ indicate—from side effects (e.g., increased epileptiform activity).^3^^,^^4^^,^^31^

Characterising these electrophysiological signatures is valuable for the pharmaco-EEG field because it provides a large-scale reference map of resting-state EEG differences associated with major psychotropic drug classes. The size and breadth of our dataset enable the detection of subtle yet consistent patterns across multiple EEG domains, including spectral power, connectivity, and nonlinear dynamics. This broad overview may help to contextualise future findings, highlight medication classes showing the most robust associations, and inform more targeted mechanistic or longitudinal investigations. By making the full results publicly available through an interactive interface, we aim to offer a practical resource for hypothesis generation, cross-study comparison, and the interpretation of EEG findings in medicated psychiatric populations.

## Methods

### Dataset

The EEG data used in this study were obtained from archival databases from two psychiatric hospitals in Warsaw, Poland: Nowowiejski Hospital for Adults and Children (NHAC) and the Institute of Psychiatry and Neurology (IPaN). Data collection spanned approximately 20 years, beginning in the early 2000s, and comprises 14,633 EEG recordings of 11,053 patients from NHAC and 20,624 recordings of 16,522 patients from IPaN.

EEG recordings from both NHAC and IPaN were acquired using the Elmiko Digitrack system, using 19 electrodes positioned according to the international 10–20 system, with a sampling frequency of either 240 or 250 Hz. For NHAC recordings, the reference electrodes were placed on the mastoids, while for IPaN recordings, either the bipolar Pz-Cz electrode or Fpz served as the reference.

During EEG acquisition, patients were seated in a dimly lit room and instructed to keep their eyes closed (EC period). They were subsequently prompted to open their eyes (EO period) or to perform deep breathing (hyperventilation, HV period). At the end of the session, photic stimulation was administered using a lamp flashing at approximately 14 Hz (PS period). The full recording lasted approximately 16–17 min, with 629 and 617 s for EC, 25 and 57 s for EO, 292 and 181 s for HV, and 103 and 130 s for PS periods on average, for NHAC and IPaN datasets, respectively.

For each patient, clinical and demographic data, including age, sex, diagnosis, and prescribed medications, were obtained from medical records (psychiatric referrals). Age and sex were self-reported in these records.

### Preprocessing

EEG data preprocessing was carried out using EEGLAB (version 2021.1). Firstly, noisy channels (kurtosis > 6SD) were identified and interpolated using spherical interpolation. Line noise (50 Hz) was attenuated with the CleanLine plugin.^41^ To unify data across the two datasets, all recordings were resampled to 250 Hz and re-referenced to a point in infinity using the Reference Electrode Standardisation Technique (REST),^42^ as this re-referencing method is more reliable for low-density montages than the common average reference or other alternatives.^43^, ^44^, ^45^

Subsequently, a high-pass filter with a passband edge of 1.5 Hz was applied to mitigate low-frequency artefacts such as those arising from electrode movement or perspiration. For the removal of noisy fragments, the Artefact Subspace Reconstruction (ASR) method was employed^46^ with window criterion of 20 SD. Additionally, to address spatially localised artefacts, channel interpolation based on the FASTER algorithm^47^ was applied. This method identifies and interpolates channels exhibiting outlier characteristics within 1-s epochs. After preprocessing, 20,586 EEG records remained from IPaN and 14,525 from NHAC, as some files were significantly artefacted (i.e., more than 50% of the epochs were considered noisy in the ASR step) and thus excluded. Table S7 shows pseudocode for the preprocessing pipeline.

Fig. S19 shows an example of representative EEG recording fragments from both hospitals, before (Fig. S19, top) and after (Fig. S19, bottom) preprocessing, and Fig. S20 shows examples of artefact handling by our preprocessing pipeline.

### Participants

In this study, only data of adult patients (age ≥ 18 years, *N* = 27,785) were used. We excluded patients with missing information about age, diagnosis, or medicines taken. At the end, 24,366 records were used for the analysis, including 11,616 from women and 12,750 from men, with the mean age *M* = 42.7 years, *SD* = 17.4, range 18–96 years. The patients were diagnosed with a wide range of neuropsychiatric disorders, with psychotic disorders and depression being the most common diagnoses. Fig. S11 shows the distribution of disorders in the dataset.

### EEG signal features

From the preprocessed data, we calculated a wide range of measures to obtain a comprehensive overview. These included spectral measures (Welch's spectrogram (1-s Hanning window, step 0.5 Hz), alpha peak frequency), connectivity measures (coherence, imaginary part of coherence (imcoh), phase slope index (psi), phase locking value (plv), phase lag index (pli), directed phase lag index (dpli), weighted phase lag index (wpli), envelope correlation) and nonlinear measures (spectral entropy, SVD entropy, Higuchi Fractal Dimension (HFD), Detrended Fluctuation Analysis (DFA), Hjorth mobility, Hjorth complexity, Vector-Autoregressive (VAR) coefficients). For the connectivity measures, we used both coherence matrices with 0.5 Hz resolution and band-wise measures; a general measure for coherence in four traditional frequency bands (theta (4–7 Hz), alpha (8–13 Hz), beta (14–28 Hz), and gamma (30–60 Hz). Spectral features were additionally normalised within patients. The features were calculated using Python 3.8.8, with mne, pyeeg, scipy.signal, and mne_connectivity packages. All these measures were calculated separately for EC, HV, and PS periods. We did not analyse the EO period, as its duration was not long enough to obtain reliable measures for some of the features. Taking together all measures, for all electrodes and frequencies, and three events, we obtained 75,132 signal features from each recording. The feature data was then z-scored, and patients whose value of any feature exceeded 20 standard deviations were removed as outliers.

### Dimensionality reduction

As the number of obtained features was vast, we decided to use Principal Component Analysis^48^ for dimensionality reduction. PCA transforms the original variables into a smaller set of uncorrelated components that capture the most variance in the data. It does so by finding new axes (principal components, PCs) along directions of maximum variance and projecting the data onto them. We retained 1560 components that collectively explain 90% of variance in the data for further analysis. Subsequently, to translate the PCA results back to the original signal features, we inspected the component loadings and identified the features that contributed most strongly to each principal component (PC) of interest. We define the most contributing features as those for which the absolute values of PCA coefficients exceeded the component-specific mean by at least 2 standard deviations. Thus, the threshold was determined separately for each PC. Values exceeding 2SD are often considered as extremes (potential outliers/unusual observations in the dataset).^49^ Under an approximately normal distribution, this heuristic also approximates a critical-value rule for identifying loadings larger than expected by chance with 95% confidence interval, proposed by Peres-Neto et al.^50^ As a sensitivity check, an alternative method for identifying the most contributing features, namely the 95th-percentile cutoff, yields similar conclusions (90% of the selected features overlapped between the two methods).

### Medicine groups

We excluded patients with missing medical data, that is, with unknown diagnosis or prescribed medicines. Then, we divided patients into groups based on the medications they were taking. We classified psychotropic medicines occurring in the database into 14 groups: Anticholinergic (*N* = 147), Benzodiazepines (BDZ, *N* = 2656), Noradrenergic and Specific Serotonergic Antidepressants (NaSSA, *N* = 1049), Serotonin antagonist and reuptake inhibitors (SARI, *N* = 515), Serotonin-Norepinephrine Reuptake Inhibitors (SNRIs, *N* = 1308), Serotonine Selective Reuptake Inhibitors and Serotonin Modulator and Stimulators (SSRIs + SMS, *N* = 2720), acetylcholinesterase inhibitor (AChE-I, *N* = 151), two types of anticonvulsants: sodium-channel blocking (AED (Na), *N* = 3771) and calcium-channel blocking (AED (Ca), *N* = 347), antipsychotic typical (N = 2901) and atypical (*N* = 5965), opioid (*N* = 257), sedative-hypnotic (*N* = 223), tricyclic antidepressant (TCA) (*N* = 112). We decided to combine the SSRI and SMS groups due to their similar mechanism of action (SMS also have affinity to the serotonin transporter, but lower than classical SSRI). Some of the patients (N = 1224) were taking medications that could not be classified as one of the above drug classes (they were not assigned to any group of interest, but they were retained in the dataset and included as an “other medications” category in the one-vs-rest (OvR) comparisons). Because some patients (N = 7087) took more than one medicine from different classes, medication groups were not mutually exclusive. Given the OvR design, we modelled drug exposure in a binary manner (presence vs absence of the drug of interest) and did not attempt to quantify the effects of multi-drug therapy. To provide transparency regarding multi-drug patterns, we include a Jaccard similarity graph summarising the most frequent class combinations (Fig. S12). It shows that benzodiazepines and sodium-channel blocking anticonvulsants are often administered together with antipsychotics. Moreover, the group characteristic distribution plots show the distributions of other medication classes across the groups (Figs. S4–S9, D). Chi-squared tests comparing the distributions of other medicines between the drug of interest group and other medicine groups show that the differences were not statistically significant (Bonferroni-Holm corrected).

We considered only the groups for which the number of patients was a minimum of 300 (BDZ, SARI, SNRI, SSRI, anticonvulsant (Na and Ca blocking), antipsychotics (typical and atypical)) as the groups of interest for the analysis in this paper, but statistics for EEG features for the other drugs can be found in our online database. There was also a group of patients not taking any psychotropic medicines (drug-naïve, *N* = 3225).

For the statistical comparisons, we used a One-vs-Rest scheme and equalised the comparison groups: for each medicine group, we created a same-size control group containing patients from other medicine groups. Patients were matched by age, sex and diagnosed disorder to the medicine group; also, the proportion of data from the two hospitals was equalised (see Figs. S13–S18), so the only differing factor between the groups was whether or not patients took the medicine of interest. We also tried to equalise the distribution of other medicines in the control group, however, this was not always possible while keeping the disorders matching, as patterns of alternate treatments occurred in some disorders (for example, typical vs atypical antipsychotics in psychotic disorders, SSRIs vs SNRIs in depression). We acknowledge this as a limitation of our study. Therefore, we performed an additional analysis, comparing the medicine groups to drug-naïve patients, also matched by age, sex, and diagnosis, whenever possible (for some drugs, for which N was exceptionally large, we undersampled the medicine group to match the N and distributions of drug-naïve patients). Moreover, we performed an additional sensitivity analysis, for which we excluded multi-drug cases. The results of this analysis are shown in Tables S1–S6. We repeated the matching 10 times (separately for OvR, drug-naïve, and single-drug comparisons), choosing a different control group each time, and we averaged the statistics’ results to obtain robust effects. Graphs comparing the age, sex, and diagnosis distributions for each group (averaged from 10 matchings), together with statistical tests results whether the distributions were equal, are shown in Figs. S4–S9 in the Supplementary Material.

### Ethics

The usage of the dataset for this research was approved by the Bioethics Committee at the Medical University of Warsaw (AKBE/281/2025). As this was a retrospective study based on existing data, the requirement for written informed consent was waived by the ethics committee. The study was conducted in accordance with the 2024 Declaration of Helsinki.

### Statistics

To compare the calculated EEG features and PCA scores (the coordinates of each patient in the obtained reduced feature space) between the groups described above (separately for One-vs-Rest and medicine vs drug-naïve comparisons), while controlling for potential site and acquisition time bias, we used the hierarchical mixed-effect regression, with medication class and year of EEG recording as independent variables and hospital site as a grouping variable. To assess group differences, we applied ANOVA with Bonferroni-Holmes correction for multiple comparisons. To reduce dependence on a single random group matching (see section Medicine groups), only features/components that met the corrected threshold in at least 7 of the 10 matchings were considered significant in the final analysis. The number of matchings in which the given component was significant is shown in Tables S1–S6. We also calculated the effect size for each comparison, using Hedges' g effect size measure (due to its lack of sensitivity to sample sizes).^51^

Fig. S10 shows the graphical summary of our methodology.

### Role of the funders

The funders had no role in the design of the study; the collection, analysis, or interpretation of the data; the writing of the manuscript; or the decision to submit the work for publication.

## Results

### Data

In this study, we leveraged a database of 24,366 resting-state clinical EEG recordings from two psychiatric hospitals in Warsaw to investigate the effects of psychotropic medications on brain electrophysiology. To minimise potential site-specific bias, the datasets were harmonised by re-referencing to infinity, resampling to 250 Hz, and performing uniform preprocessing (see Methods: Preprocessing for details). We separated patients into groups, according to classes of psychotropic medicines they were taking (BDZ [*N* = 2531], NaSSA [*N* = 996], SARI [*N* = 473], SNRI [*N* = 1132], SSRI + SMS [*N* = 2538], sodium channels blocking anticonvulsants (AED Na) [*N* = 2962], calcium channels blocking anticonvulsants (AED Ca) [*N* = 303], atypical AP [*N* = 5944], typical AP [*N* = 2721]) and compared EEG signal features between each of those groups and other patients (One-vs-Rest analysis) and against drug-naïve patients [*N* = 2771]. For the analysis, we balanced the comparison groups by age, sex, and especially diagnosis, to eliminate confounding factors. Our results primarily focus on the One-vs-Rest comparison, as this analysis controls for the placebo effect (in contrast to the naive group) and allows for better group balancing (see Fig. S4–S9).

EEG signal may be described by diverse quantitative measures, such as spectral features (power of different frequencies in the signal), connectivity measures revealing relationships between the electrode pairs, and nonlinear measures, quantifying signal complexity. Here we computed a wide spectrum of these features, including power spectra (1-s Hanning window, step 0.5 Hz), coherence (6-s window, step 0.5 Hz (method 1) and in commonly used frequency bands [theta (4–7 Hz), alpha (8–13 Hz), beta (14–28 Hz) and gamma (30–60 Hz)]; method 2]), phase locking value (6-s window, commonly used frequency bands as stated above) and other phase synchronisation measures, spectral entropy, Higuchi fractal dimension and other signal complexity measures. For a complete list, please refer to the methods. Overall, we computed 75,132 features during eyes closed (EC), deep breathing (hyperventilation, HV), and photostimulation (PS). Below, we present our analysis and interpretation of the results, alongside an accompanying online database that constitutes a key contribution of this paper, containing all statistical comparisons and descriptive statistics.

**Fig. 2:**
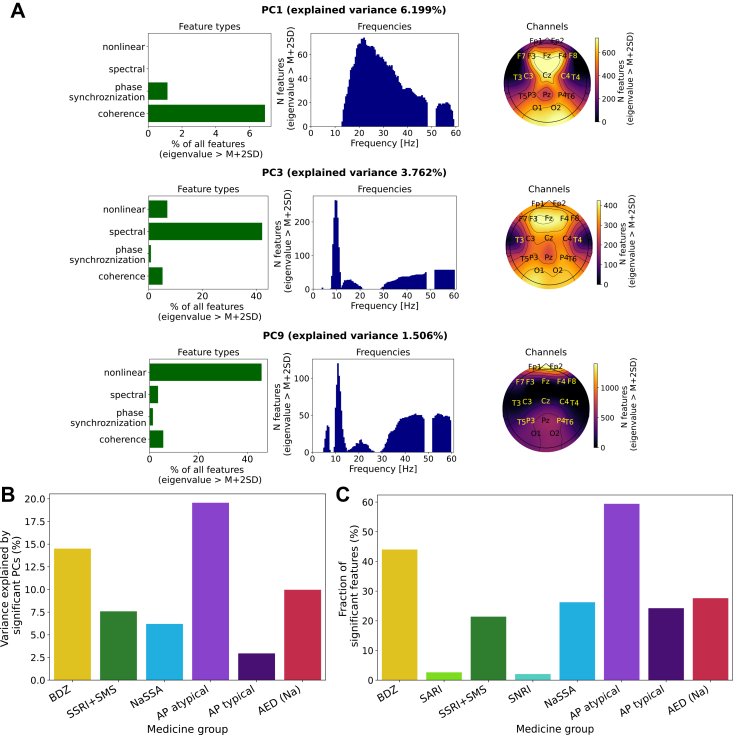
Visualisation of exemplificatory PCA components and main effects in our analysis. **A.** Features contributing the most (with eigenvalues higher than the mean eigenvalue by at least 2 SD) to several representative PCs. We are presenting the distribution of features depending on different domains: left, based on the type of analysis; middle, for different spectral contributions; and right, for scalp localisation. We can observe aggregation in each domain. **B.** Percentage of variance in the data explained by the significant PCs for the medicine groups. **C**. Percentage of significant features (in the single-feature analysis) for the medicine groups (FDR corrected).

Using a broad array of EEG characteristics is crucial for a more complete understanding of how drugs impact brain activity. This, however, creates a high-dimensional feature space, which is difficult to interpret. To address this, we applied Principal Component Analysis (PCA), a widely used dimensionality reduction technique that transforms correlated variables into a set of uncorrelated components.^48^ PCA enables us to reduce redundancy among features and retain the most informative aspects of the data, thereby simplifying downstream analyses while preserving key physiological patterns. Here, we applied PCA to the entire dataset (*N* = 24,366 patients, after excluding outliers and missing data) using all computed features. We retained 1560 components, explaining 90% of total variance in the data. The resulting principal components (PC) effectively captured interdependencies among features, organising them into physiologically coherent spatial and frequency patterns. For instance, PC1 (Fig. 2A, top) encompasses solely connectivity features, mainly in beta frequency range, reflecting coordinated oscillatory activity across areas. PC3 (Fig. 2A, middle) exhibited a pronounced frequency peak in the alpha frequency band, grouping predominantly spectral features. Alpha is the most prominent EEG rhythm, often readily visible in raw recordings and characterised by a distinct peak in the resting-state power spectrum. PC9 (Fig. 2A, bottom) comprised predominantly nonlinear features over frontal and parietal regions, mainly in the alpha and gamma ranges.

These patterns indicate that the PCA components reflect meaningful neurophysiological structure, rather than arbitrary statistical variance. Moreover, most components displayed a pronounced alpha peak in their frequency eigenvalue profiles—a hallmark of resting-state EEG and a strong indicator of physiological validity.

Fig. 2A shows features, electrodes, and frequencies contributing the most to the several above-mentioned exemplificatory PCA components. See Fig. S1 for the top 10 components, in terms of explained variance. We consider here (and in all further analysis) features that have eigenvalues (loadings) higher than the mean by at least two standard deviations in a given PCA as “the most contributing features”.

After the dimensionality reduction step, we tested whether principal component scores differed between the medication groups matched as described above. These scores represent each patient's coordinates in the principal-component space. PCA was performed once on the full dataset, and the resulting component scores were then used as outcome variables in a mixed-effects hierarchical regression model that controlled for site and time of data collection (see Methods). Group effects were subsequently evaluated using ANOVA. Our analysis revealed significant differences in PCA components between patients using BDZ, SSRIs, NaSSA, APs, and Anticonvulsants blocking sodium channels vs other medicines and drug-naïve patients. Fig. 2B shows the percentage of variance in the data explained by those significant components. No significant PCs were found for other medicine groups (although for SNRI and SARI, some single features distinguished them from other medicine groups, Fig. 2C). Comparisons for PCs were Bonferroni-Holm corrected, while for single features FDR corrected (as we considered Bonferroni-Holm too conservative for over 75,000 comparisons). Below, we discuss the effects for specific groups.

### Benzodiazepines (BDZ)

Fifteen principal components distinguished patients using BDZs from other medication groups, jointly explaining 14.51% of the total variance in the data. The components showing the largest effect sizes in the OvR comparison were PC3 (p < 0.001, g = 0.34, 95% CI [0.28, 0.39]), PC11 (p < 0.001, g = 0.29, 95% CI [0.23, 0.34]), PC27 (p < 0.001, g = 0.19, 95% CI [0.13, 0.24]), and PC6 (p < 0.001, g = 0.16, 95% CI [0.11, 0.22]). Twenty-five PCs differentiated people using BDZs from drug-naïve participants. For more detailed results for these contrasts, see Table S1.

**Fig. 3:**
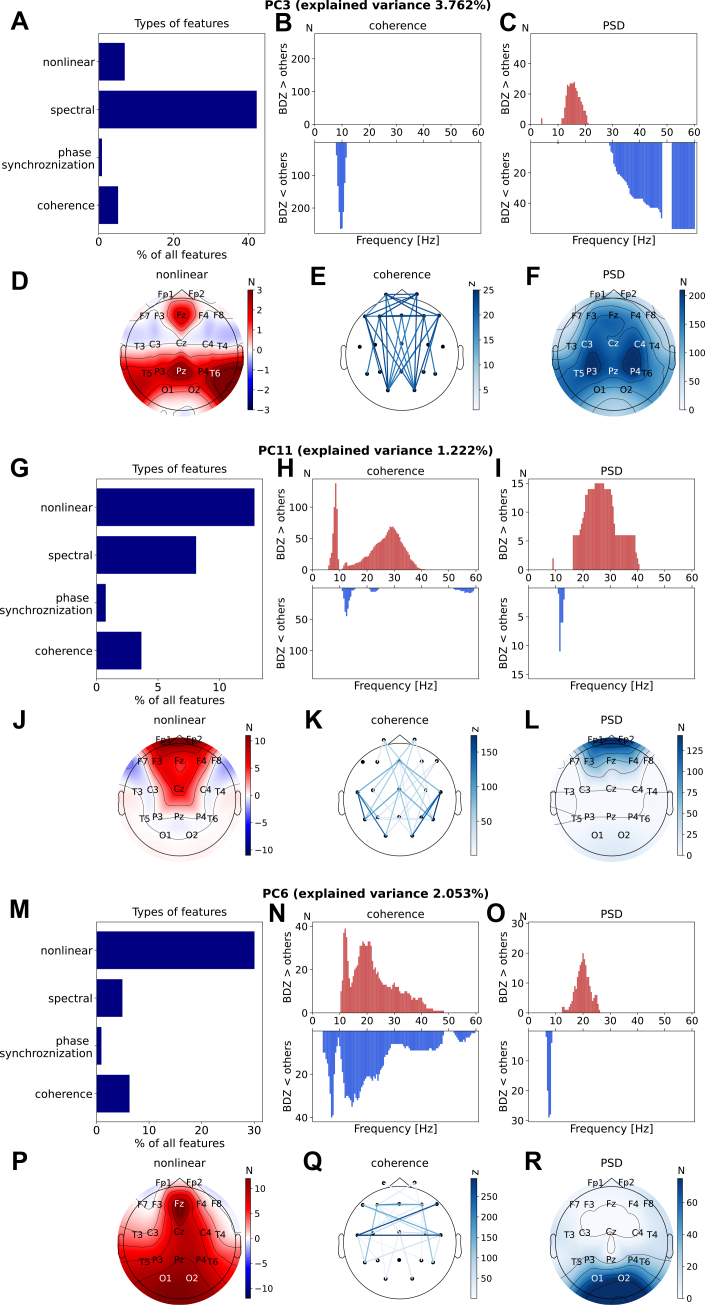
Three PCs with the highest effect sizes and explaining the most variance in BDZs vs other medications [PCs 3,11 and 6]. **A, G,** and **M** show the percentage of eigenvalues from each type of EEG features. **B**, **C, H, I, N, O** represent the number of features as a function of frequency, separately for connectivity measures (**B, H, N**) and power spectral density (PSD, **C, I, O**). The increase of feature values in the BDZ group, as compared to other drugs, is marked in red, while the decrease is marked in blue. The topomaps show the number of features as a function of localisation, separately for nonlinear features (**D, J, P**), connectivity measures (**E, K, Q**), and PSD (**F, L, R**). For nonlinear features, the increase of feature values in the BDZ group is shown in red, while the decrease is shown in blue. For PSD, an absolute value is shown. For connectivity, 30 connections with the highest number of contributing features are shown.

PC3 was dominated by spectral features (Fig. 3A), characterised by increased low-beta power and decreased gamma power in patients treated with BDZ (Fig. 3C). It also included nonlinear features predominantly localised to the right temporo-parietal region (Fig. 3D) and decreased alpha coherence (Fig. 3B). PC11 was mainly composed of nonlinear and spectral features (Fig. 3G) in frontal areas (Fig. 3J and L), showing increased beta and reduced alpha power (Fig. 3I) in the BDZ group. This component further reflected elevated low-alpha and beta coherence in patients undergoing BDZ treatment (Fig. 3H). In PC6, nonlinear features contributed most strongly (Fig. 3M), indicating increased whole-brain complexity in patients using BDZ (Fig. 3P). PC6 also encompasses connectivity features, with decreased theta-low/alpha while increased high-alpha synchronisation, and with both increases and decreases in beta-band synchronisation (Fig. 3N), predominantly in the fronto-central area (Fig. 3Q). Spectral features within PC6 were characterised by elevated beta and reduced low-alpha power (Fig. 3O), mainly in occipital regions (Fig. 3R). PC27 (Table S1) was dominated by connectivity features (both coherence and phase synchronisation). It shows an increase in low-alpha and gamma connectivity and a decrease in theta, high-alpha, and beta connectivity. Table S1 summarises findings from all significant PCs.

**Fig. 4:**
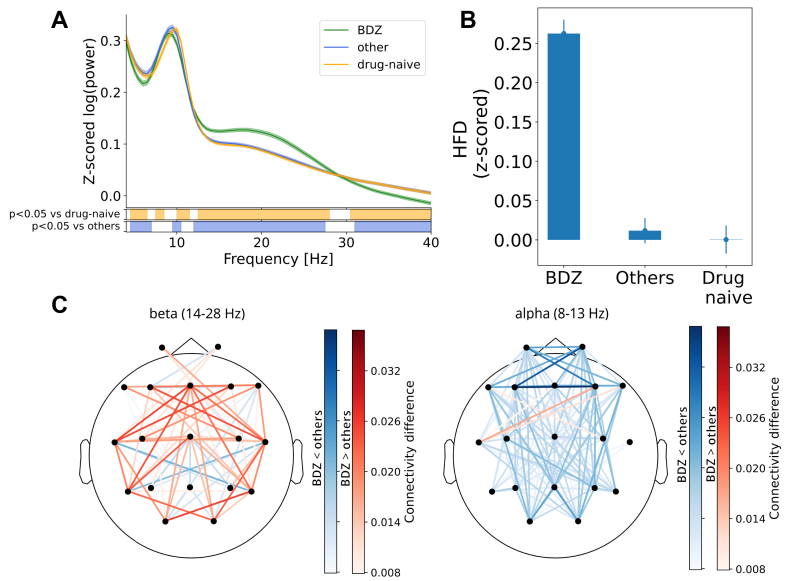
The most contributing features to the PCs that differentiate patients treated with BDZs from individuals taking other medicines. **A.** Mean logarithm of z-scored power spectrum density for the frontal electrodes (which had high eigenvalues in both PC6 and PC11) for people using BDZs vs other medicine groups and drug-naïve patients during eyes closed condition. The graph shows an increase in beta power (14–28 Hz) and a decrease in gamma power (>30 Hz) (and, to a lesser extent, a decrease in theta (4–7 Hz) power and in alpha power at the peak (∼10 Hz) in BDZ group compared to the other groups). The coloured bar at the bottom shows whether the difference was significant (FDR-corrected) between individuals receiving BDZs and drug-naïve patients (yellow) and other drug classes (blue). **B.** Mean Higuchi Fractal Dimension (HFD) from the frontal electrodes (the most contributing to PC11) during eyes closed condition. It shows an increase in the signal complexity in patients taking BDZs vs the other groups. **C.** Connectivity difference between people using BDZs and other medicine groups during eyes closed in the beta frequency range (left) and the alpha frequency range (right). The increase in coherence is marked with red lines, while the decrease is marked with blue lines, and the intensity of the colour reflects the magnitude of the difference. Only significantly differing connections are shown (FDR-corrected). The graph shows mostly an increase in beta coherence and predominantly a decrease of coherence in alpha band in the BDZ group.

When analysing features directly, we can observe differences in the features that load onto the described PCs. Fig. 4 presents several significant feature effects of interest: spectral power for the electrodes most contributing to PC3 and PC11 (Fig. 4A), mean Higuchi Fractal Dimension (HFD, a signal complexity measure) from electrodes most contributing to PC11 (Fig. 4B), alpha and beta coherence (Fig. 4C), which were contributing to most of the significant PCs.

Power spectrum analysis (Fig. 4A) revealed significant effects in beta and gamma power. Beta power was significantly higher and gamma power was significantly lower for all brain areas for patients using BDZs than for other medicine groups and for drug-naïve patients, consistently with PC3 (Fig. 3C). Smaller but significant differences in the power are also visible in theta and alpha ranges, which were decreased in people treated with BDZs (Fig. 4A). This is consistent with a decrease in theta/low-alpha power suggested by PC6 (Fig. 3O) and also by PC33 and 37 (Table S1). Complexity comparison (Fig. 4B) indicates increased signal complexity in the BDZ group vs other groups, which matches effects indicated by PCs 3, 6, and 11 (Fig. 3D–J, P). Connectivity patterns were more complex, several PCs showing both increase and decrease in a wide range of frequencies. Direct analysis of alpha and beta coherence (Fig. 4C) helps to disentangle these effects, showing a decrease in alpha coherence, except for several fronto-central connections, and mostly an increase in beta coherence, except for centro-parietal areas, in the BDZ group.

To explore all significant effects please refer to our interactive database at https://brainwavesrx.nencki.edu.pl/. This website contains all statistical effects for all medications groups with visualisation tools.

The majority of the PCs that significantly distinguish patients using BDZs from other medicine groups incorporated features in beta frequency, particularly, indicating elevated beta power. This is in accordance with the literature, as the increase in beta power is the most consistent effect of benzodiazepine administration on brain waves.^5^^,^^24^, ^25^, ^26^, ^27^, ^28^ Beta power increase is considered a quantitative biomarker for GABA_A_ receptor modulation^52^ and it correlates with subject-rated decreases in anxiety.^39^

Theta, alpha and gamma power, and nonlinear features also distinguished people treated with BDZs from other medicine groups and from drug-naïve subjects. We replicated the results showing the increase of beta power in the BDZ group, as mentioned above, and we showed an additional decrease in gamma, alpha and theta power (Fig. 4A). The decrease of alpha power effect in patients using BDZs vs drug-naïve subjects is also reported in several studies.^25^^,^^28^ The increase in signal complexity in BDZ group is consistent with the work of Michail et al.,^25^ reporting an increase in fractal dimension after alprazolam administration.

The feature contributing to the differences between people receiving BDZ treatment and other patients, also often indicated in different PCs along with beta power, was coherence, mainly in alpha and beta frequency bands. PCs 6 and 11 encompass beta connectivity features mostly from occipital and fronto-central regions, where an increase in beta coherence was observed in the BDZ group. Similarly, Romano-Torres et al.^53^ report an increase in interhemispheric coherence in the high-beta range after diazepam administration. For the alpha connectivity, PCs 3 and 11 show contrastive effects (a decrease in long-range connectivity in PC3 and an increase in short-range connectivity in PC11), while PC6 shows a decrease in low-alpha connectivity and an increase in high-alpha connectivity. Single-feature coherence analysis (averaged across the whole alpha band) is more consistent with decreased alpha coherence in patients taking BDZs, although some short-range connections show an increase (Fig. 4C, right). In the same study of Romano-Torres et al.,^53^ interhemispheric high-alpha coherence was decreased.

### SSRI + SMS

**Fig. 5:**
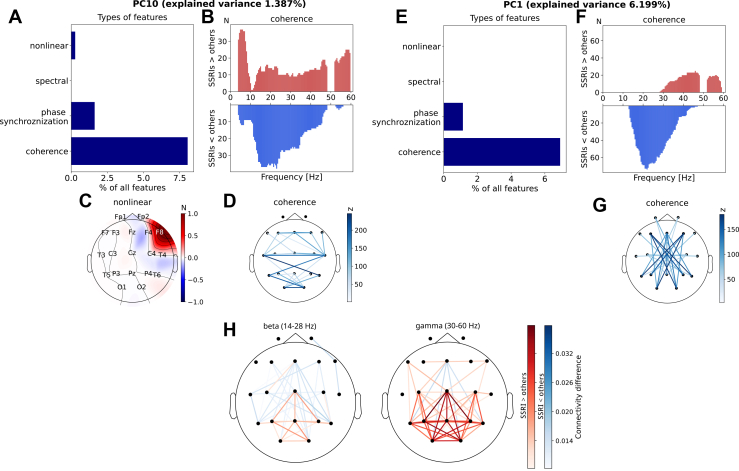
The PCA components that significantly distinguish people treated with SSRIs from other medicine groups, and the coherence features that contribute the most to them. **A, E**. The percentage of eigenvalues from each type of EEG features. **B, F.** The number of features as a function of frequency for connectivity measures. The increase of feature values in the SSRI group, as compared to other drugs, is marked in red, while the decrease is marked in blue. **D, G**. Connections between electrode pairs with the highest number of contributing features. **H**. Connectivity difference between the SSRI group and other groups in the frequency ranges that contribute to these PCs the most, i.e., beta (left) and gamma (right). Only significant connections are marked. Blue colour indicates the decrease of coherence in the SSRI group, while red indicates the increase.

When analysing the impact of SSRIs and SMS on EEG activity, we found that two PCs, PC10 (p < 0.001, *g* = 0.18, 95% CI [0.12, 0.23]) and PC1 (p < 0.001, *g* = 0.17, 95% CI [0.12, 0.23]), significantly differentiated them from other medicine groups. Both PCs encompass connectivity features in beta and gamma frequencies. Beta coherence was mostly decreased (Fig. 5B and F), while gamma coherence was increased (Fig. 5B and F) for subjects taking SSRIs. Additionally, PC10 indicates an increase in theta/low-alpha connectivity (Fig. 5B). For comparison with drug-naïve subjects, 3 PCs were significant, including both PC10 and PC1 (Table S2).

Only connectivity differentiates people treated with serotonergic drugs from other medicine groups. We observed an increased gamma coherence (Fig. 5B, F and H) and mostly decreased beta coherence in patients taking SSRIs and SMS (Fig. 5B, F and H). Similarly, Nissen et al.^12^ reported an increase in gamma connectivity (specifically, phase-lag index) and a decrease in temporal beta connectivity after escitalopram and vortioxetine administration in healthy males. Other studies examining connectivity have not treated SSRIs as a separate class, but instead have grouped them within the broader category of antidepressants. These studies reported higher beta-band connectivity in the antidepressant group compared with other drug classes.^54^^,^^55^ Beta-band functional connectivity (assessed by graph measures) also differentiated responders from non-responders of antidepressant (mainly SSRI) treatment.^56^ Other studies on SSRI EEG effects report mostly alterations in spectral features,^5^^,^^10^, ^11^, ^12^, ^13^ often using polysomnography.^57^^,^^58^ We did not find significant components that gather spectral features for this medication class.

Interestingly, for PC1, we also observed a significant effect (p < 0.001, g = 0.38, 95% CI [0.30, 0.47], Table S3) for patients taking NaSSA, but the effect was in the opposite direction–beta connectivity was increased, while gamma connectivity decreased in the NaSSA group. NaSSA, likewise SSRIs, increase serotonergic transmission, with additional noradrenergic transmission elevation. This may suggest that this component was associated with the activity of serotonergic pathways.

### Antipsychotics (AP)

**Fig. 6:**
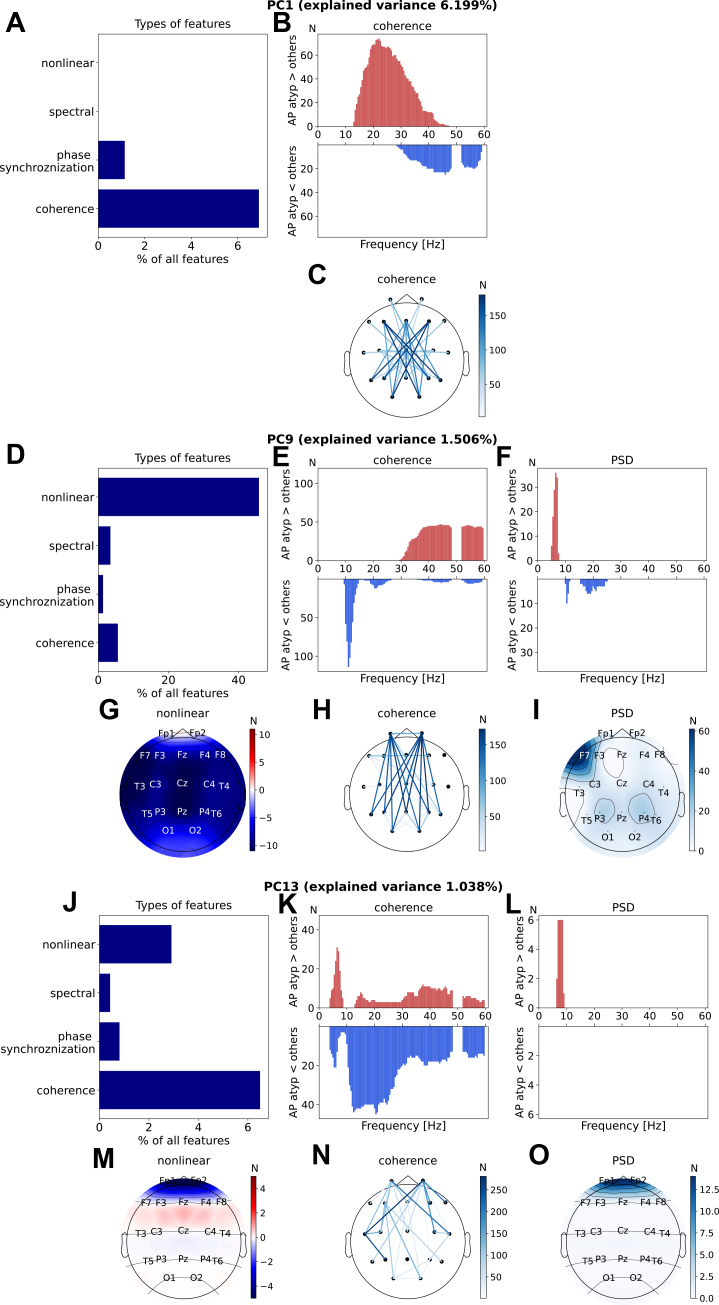
The PCA components that significantly distinguish between the atypical AP group and other medicine groups, with the highest effect sizes. **A, D, J.** The percentage of high eigenvalues from each type of EEG features. **B, E, F, K, L** represent the number of features as a function of frequency, separately for connectivity measures (**B, E, K**) and PSD (**F, L**). The increase of feature values in the AP group, as compared to other drugs, is marked in red, while the decrease is marked in blue. **C, G–I** and **M–O** show the number of features as a function of localisation, separately for connectivity measures (**C, H, N**), PSD (**I, O**) and nonlinear features (**G, M**). For nonlinear features, the increase of feature values in the AP group is shown in red, while the decrease is shown in blue. For PSD, an absolute value is shown. For connectivity, 30 connections with the highest number of contributing features are shown.

Fifteen PCA components significantly distinguished patients taking atypical antipsychotics from the other drug classes, collectively explaining 19.56% of the total variance (Table S4). The components with the largest OvR effect sizes were PCs 1 (p < 0.001, g = 0.27, 95% CI [0.24, 0.31]), 9 (p < 0.001, g = 0.18, 95% CI [0.15, 0.22]), and 13 (p < 0.001, g = 0.18, 95% CI [0.14, 0.22]), as shown in Fig. 6. Seventeen PCs were significant when compared to drug-naïve subjects.

**Fig. 7:**
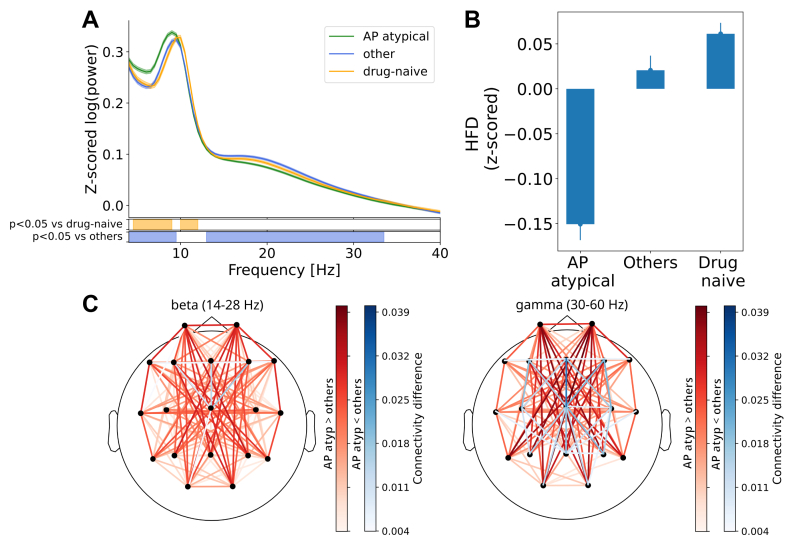
Features contributing the most to the PCs that significantly distinguish between atypical AP and other medicine groups. **A.** Mean logarithm of z-scored power spectrum density for the frontal electrodes (the most contributing to PCs 9 and 13) for people using atypical APs vs other medicine groups and drug-naïve patients during eyes closed condition. The graph shows that the spectrum of patients taking antipsychotics is shifted to the left, as compared to the other groups. The coloured bar at the bottom shows whether the power difference was significant (FDR-corrected) between people using atypical APs and drug-naïve patients (yellow) and other drug classes (blue). **B.** Mean z-scored HFD for the frontal electrodes, which had high contributions to several significant PCs, during photostimulation. The AP atypical group exhibits reduced signal complexity compared to the other groups. **C**. Coherence differences between the AP atypical group and other medicines during eyes closed. Only significantly differing connections are shown (FDR-corrected). Blue colour indicates the decrease of coherence in the AP atypical group, while red indicates the increase. Both beta (left) and gamma (right) coherence was predominantly increased in the AP atypical group.

Signal complexity was the feature most consistent in the differences between the atypical antipsychotic group and other medication groups, appearing in 13 out of 15 significantly different components. Two out of three PCs with the highest effect sizes contained features indicating a reduction in EEG signal complexity among patients taking atypical antipsychotics (Fig. 6G–M). Other key differentiating features in patients using atypical antipsychotics included alpha, beta, and gamma coherence (as indicated by PCs 1, 9, 13, Fig. 6 B, E, K and Fig. 7C). Additionally, atypical antipsychotic use was associated with elevated theta/low-alpha spectral power (Fig. 6F and L) captured by PCs 9 and 13, and reduced beta power, indicated by PCs 6, 18, 29, 36 and 93 (Table S4) and Fig. 7A (spectral features).

Further analysis of the spectral features present in the above-mentioned PCs revealed that the EEG spectrum of patients treated with antipsychotics was shifted toward lower frequencies (Fig. 7A), indicating overall EEG slowing — an effect previously reported.^3^^,^^4^^,^^7^^,^^30^^,^^31^ In terms of power differences, this slowing manifests as increased theta and low-alpha power, decreased high-alpha power (reflecting a leftward-shifted alpha peak), and reduced beta power–the same set of features that contributed most strongly to PCs significantly differentiating the atypical AP group.

Analysis of complexity measures, exemplified by Higuchi Fractal Dimension, confirmed that people using atypical APs exhibited reduced signal complexity relative to other groups (Fig. 7B). Connectivity analyses also revealed group differences: beta coherence was increased in the atypical antipsychotic group (Fig. 7C, left), as suggested by PC1 (Fig. 6B), and gamma coherence showed a similar pattern of elevation (Fig. 7C, right), in accordance with what PC9 shows (but in contrast to PC1). Possibly, the inconsistency between those two PCs indicates increased long-range gamma coherence and decreased short-range coherence, as most of the connections with the highest loadings in PC9 were long-range, between frontal and occipital areas (Fig. 6H), while PC1 encompasses mostly local coherence in central areas (Fig. 6C). Fig. 7C also indicates that several short-range connections within the central area exhibit decreased gamma coherence.

Fig. 7 illustrates selected effects observed in patients using antipsychotics, including spectral power differences (A), fractal dimension (B), and beta and gamma coherence (C).

**Fig. 8:**
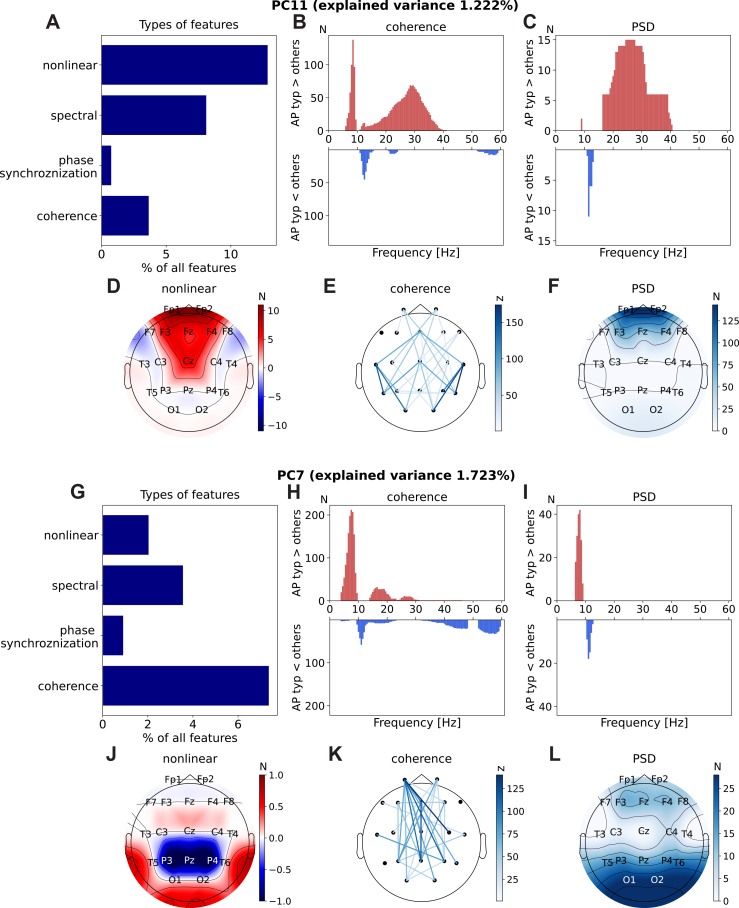
The PCA components that significantly distinguish between the typical AP group and other medicine groups. **A, G.** The percentage of high eigenvalues from each type of EEG features. **B, C, H, I** represent the number of features as a function of frequency, separately for connectivity measures (**B, H**) and PSD (**C, I**). The increase of feature values in the AP group, as compared to other drugs, is marked in red, while the decrease is marked in blue. **D–F** and **J–L** show the number of features as a function of localisation, separately for connectivity measures (**E, K**), PSD (**F, L**), and nonlinear features (**D, J**). For nonlinear features, the increase of feature values in the AP group is shown in red, while the decrease is shown in blue. For PSD, an absolute value is shown. For connectivity, 30 connections with the highest number of contributing features are shown.

**Fig. 9:**
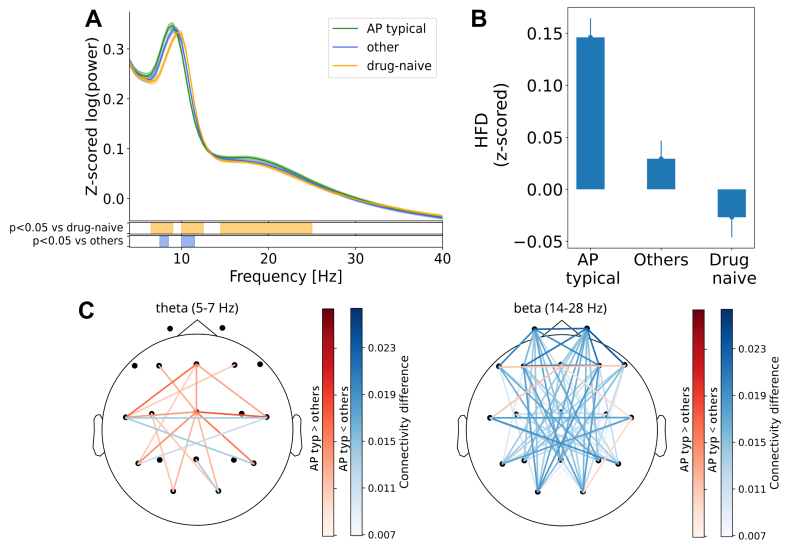
Features contributing the most to the PCs that significantly distinguish between typical AP and other medicine groups. **A.** Mean logarithm of z-scored power spectrum density for the frontal electrodes (the most contributing to PC11) for people taking typical APs vs other medicine groups and drug-naïve patients during the eyes closed condition. The graph shows that the spectrum of patients taking antipsychotics is shifted to the left, as compared to the other groups. The coloured bar at the bottom shows whether the power difference was significant (FDR-corrected) between people using typical APs and drug-naïve patients (yellow) and other drug classes (blue). **B.** Mean z-scored HFD for the frontal electrodes (the most contributing to PC11), during photostimulation. The AP typical group exhibits elevated signal complexity compared to the other groups. **C**. Coherence differences between the AP typical group and other medicines during eyes closed. Only significantly differing connections are shown (FDR-corrected). Blue colour indicates the decrease of coherence in the AP typical group, while red indicates the increase. Theta (left) connectivity was predominantly increased in the AP typical group, consistent with what PCs 7 and 11 suggest, while beta coherence (right) was mostly decreased, although in local occipital-temporal connections (right), it was increased, in accordance with PC11.

Two PCs were significant for typical antipsychotics, namely PC11 (p < 0.001, *g* = 0.17, 95% CI [0.11, 0.22]) and PC7 (p = 0.001, *g* = 0.14, 95% CI [0.09, 0.19]). The features that contribute the most to these PCs include signal complexity, increased in the typical AP in frontal (PC11, Fig. 8D) and occipital (PC7, Fig. 8J) areas, but decreased in parietal areas (Fig. 8J); increased low-alpha and beta connectivity (Fig. 8B–H); increased low-alpha but decreased high-alpha power (Fig. 8C–I) and increased frontal beta power (PC11, Fig. 8C). The power changes (Fig. 9A), similarly to the atypical group, indicate general spectral slowing. However, signal complexity (Fig. 9B) is affected in the opposite direction in the two antipsychotic treatment groups–increased after typical APs but decreased after atypical APs administration.

The EEG differences in theta and low-alpha band power observed in patients undergoing antipsychotic therapy (as indicated by PCs 7, 9 and 13, Fig. 6F and L and Fig. 8I, also shown in Figs. 7A and 9A) align well with existing literature, which consistently reports an increase in slow-wave power following antipsychotic treatment.^3^^,^^4^^,^^7^^,^^30^^,^^31^ This slowing is a direct effect of dopamine receptors blockade,^59^^,^^60^ which is a principal mechanism of action of antipsychotic drugs.^29^ In line with this, theta-power increases correlate with reductions in psychotic symptoms.^37^^,^^38^ Similarly, decreased signal complexity, visible in PCs 9 and 13 (Fig. 6G,M and Fig. 7B) after atypical antipsychotics treatment was shown previously.^61^ In a study by Takahashi et al.,^61^ treatment-naïve patients with schizophrenia exhibited higher complexity than healthy control subjects. This effect was attenuated by antipsychotic treatment, particularly in fronto-centro-temporal brain regions (which were also the regions from which the most features contributed to PCs 9 and 13 (Fig. 6G,M). Taken together, the increase in theta power and the decrease in complexity (visible in atypical APs) are compatible with drug-induced dampening of cortical excitability and stabilisation of large-scale oscillatory dynamics — mechanistic changes linked to reductions in aberrant salience and hallucinations.^62^, ^63^, ^64^ Interestingly, for typical APs, we observed mostly an increase in signal complexities, which could suggest that signal complexity and beta power are sensitive to differences between the two antipsychotic groups, likely in serotonergic pathway modulation.

The effects on higher-frequency power are less clear. In our study, atypical antipsychotics were associated with increased beta power, whereas typical antipsychotics were associated with decreased beta power. Findings in the literature are inconsistent and often depend on the specific compound and treatment duration. In general, acute administration of haloperidol, a typical antipsychotic, tends to increase beta power, whereas prolonged treatment is associated with its reduction.^65^, ^66^, ^67^ A similar pattern has been reported for clozapine,^68^ an atypical antipsychotic. By contrast, risperidone has been associated primarily with increased beta power,^30^^,^^69^ whereas olanzapine has been reported to decrease beta power.^7^ Thus, our findings are consistent with those reported for prolonged haloperidol treatment and risperidone, but not with those described for clozapine or olanzapine.

Significantly less is known about the effects of these drugs on EEG connectivity, an important feature in PCs 1, 7 and 13. Several studies suggest that schizophrenia is associated with diminished gamma-band synchronisation.^70^, ^71^, ^72^ Therefore, the observed increase in gamma coherence among patients taking atypical antipsychotics (visible in PC9, Fig. 6E, and in Fig. 7C) may reflect a normalisation of oscillatory network activity facilitated by this drug therapy. Note that in the control group, there was the same proportion of patients with schizophrenia as in the AP group. A similar effect was shown by Alegre et al.,^40^ but in response to auditory tones. They found a reduced amplitude and inter-trial phase coherence of the auditory response in the gamma range in drug-naïve patients with schizophrenia compared to controls. In patients treated with atypical antipsychotics, the response in the 30–50 Hz range was normalised to values similar to those of the control group.

Concerning connectivity in other frequencies, the literature on humans is quite scarce. Zandstra et al.^54^ showed that antipsychotic use was associated with lower alpha-band connectivity, measured by amplitude envelope correlation. Similarly, in our data, PC9 suggests a decrease in alpha coherence in the atypical AP group. EEG connectivity may also be a predictor of treatment response to APs. A decrease in theta coherence after clozapine treatment was observed in high-responders, and a decrease in beta coherence was observed in low-responders.^38^ Alpha coherence was decreased in both groups.

### Anticonvulsants

In the analysis we split anticonvulsant drugs into two classes: sodium channel-blocking [AED (Na)] and calcium channel-blocking [AED (Ca)], as these groups act through distinct neural pathways. Here, we present the results for the more common type of anticonvulsants, i.e., sodium-blocking. No significant effects were observed for calcium channel-blocking anticonvulsants.

**Fig. 10:**
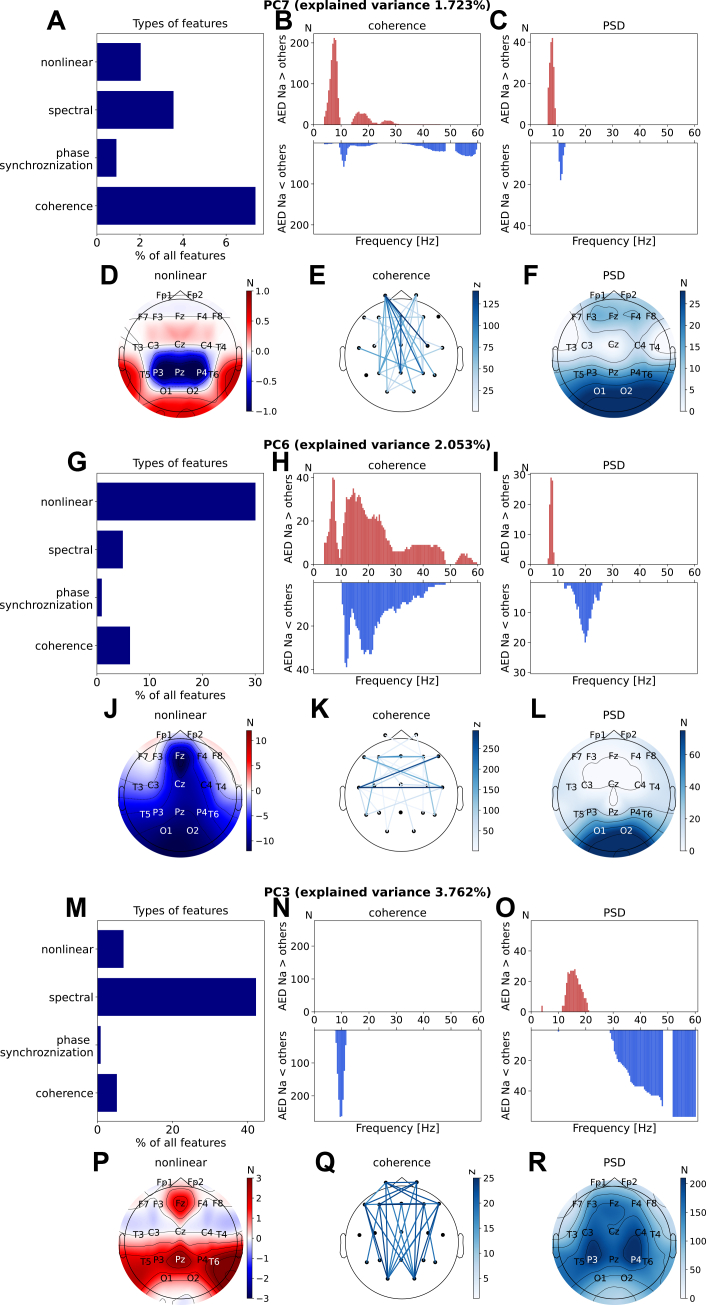
PCA components significantly distinguishing the sodium channel-blocking anticonvulsant group from the other medicine groups with the highest effect sizes. **A, G, M.** The percentage of high eigenvalues from each type of EEG features. **B, C, H, I, N** and **O** represent the number of features as a function of frequency, separately for connectivity measures (**B, H, N**) and PSD (**C, I, O**). The increase of feature values in the AED (Na) group, as compared to other drugs, is marked in red, while the decrease is marked in blue. **D–F**, **J–L** and **P–R** show the number of features as a function of localisation, separately for connectivity measures (**E, K, Q**), PSD (**F, L, R**), and nonlinear features (**D, J, P**). For nonlinear features, the increase of feature values in the AED group is shown in red, while the decrease is shown in blue. For PSD, an absolute value is shown. For connectivity, 30 connections with the highest number of contributing features are shown.

Seven components significantly differentiated patients on the sodium channel–blocking therapy from the other medication group. The highest effect sizes were observed for PCs 7, 6 and 3. PC7 (p < 0.001, g = 0.21, 95% CI [0.17, 0.26]) primarily comprised connectivity and spectral features (Fig. 10A), indicating increased theta/low-alpha power (Fig. 10C) and coherence (Fig. 10B). PC6 (p < 0.001, g = 0.18, 95% CI [0.14, 0.23]) was driven mainly by nonlinear features (Fig. 10G–J), suggesting reduced signal complexity in people using AED (Na). PC3 (p < 0.001, g = 0.13, 95% CI [0.08, 0.17]) was dominated by spectral features (Fig. 10M) and reflected increased beta power together with decreased gamma power (Fig. 10O).

**Fig. 11:**
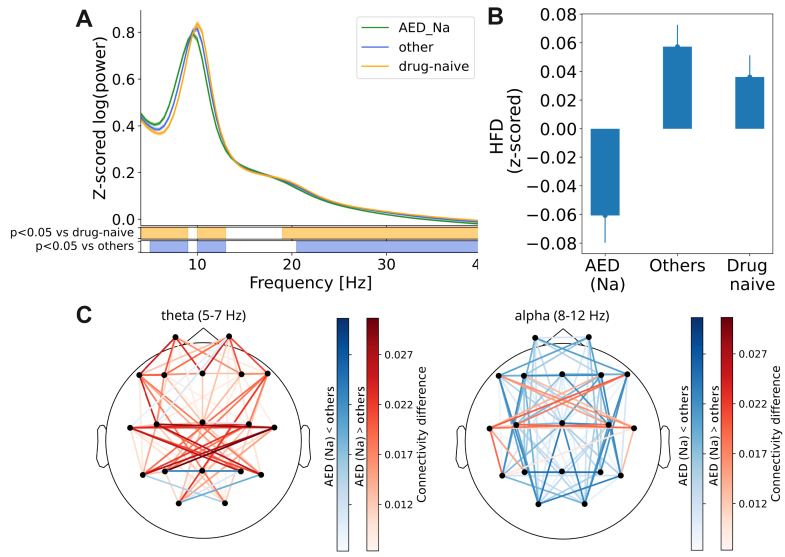
Features contributing the most to the PCs that differed significantly between patients on sodium channel-blocking anticonvulsant treatment and other medicine groups. **A.** Mean logarithm of z-scored power spectrum density for the occipital electrodes (the most contributing to PCs 6 and 7) for people using AED (Na) vs other medicine groups and drug-naïve patients during eyes closed condition. The graph shows that the spectrum of patients taking AEDs is shifted to the left, as compared to the other groups. Moreover, alpha power was decreased in the AED (Na) group. The coloured bar at the bottom shows whether the power difference was significant (FDR-corrected) between people treated with AED (Na) and drug-naïve patients (yellow) and other drug classes (blue). **B.** Mean z-scored HFD for parietal electrodes, which had high contributions to PCs 3, 6, and 7. Signal complexity was decreased for patients taking anticonvulsants, in comparison to the other groups. **C.** Connectivity differences between patients using sodium channel-blocking AED and other medicine groups during eyes closed in theta (left) and alpha (right) frequency bands. Only significantly differing connections are shown (FDR-corrected). Theta coherence was predominantly increased in the AED (Na) group, as PCs 6 and 7 suggest. Alpha coherence was predominantly decreased in AED (Na) group vs other medicines, as PC3 suggests.

Analysis of individual features confirmed effects evident in PCA. The power spectrum of patients using anticonvulsants was shifted to the left (Fig. 11A), resulting in increased theta/low-alpha power and decreased high-alpha and beta power, consistent with the patterns captured by PCs 6 and 7 (Fig. 10C–I). In addition, gamma power was reduced in patients using AED (Na), as indicated by PC3 (Fig. 10O). Complexity measures were decreased in the parietal region in the AED group (Fig. 11B), in line with PC7 and PC6 (Fig. 10D–J). Connectivity analysis (Fig. 11C) reveals predominantly increased theta coherence and mostly decreased alpha coherence in the AED (Na) group. This is reflected in increased theta/low-alpha connectivity shown by PCs 6 and 7 (Fig. 10B–H), and in decreased high-alpha connectivity shown by PCs 6 and 3 (Fig. 10H and N).

The increase in theta/low-alpha frequency power and decrease in high-alpha power in the sodium-channel blocking anticonvulsant group (indicated by PC6, PC7, and Fig. 10A) are well in line with existing studies.^34^, ^35^, ^36^ Less is known about signal complexity in patients taking anticonvulsant drugs. According to Colominas et al.,^73^ EEG complexity is decreased in patients with epilepsy compared to healthy controls, and anticonvulsant treatment normalises it by increasing the signal complexity. Our findings, however, suggest a more complex pattern, with some PCs indicating increased and others decreased signal complexity in the AED group. In particular, measures such as HFD were increased in frontal regions, but decreased in parietal and occipital regions. Dependence of AED-induced signal complexity changes on localisation may be supported by Lehnertz and Elger, who found a significant inverse relationship between carbamazepine serum level and local complexity, spatially restricted to the primary epileptogenic area, using intracranial EEG recordings.^74^ Collectively, increased low-frequency power and reduced signal complexity may serve as electrophysiological correlates of a shift in the excitation–inhibition balance toward inhibition.^75^^,^^76^ This shift reflects a principal mechanism of action of anticonvulsant drugs, which either enhance GABAergic inhibition or attenuate glutamatergic excitation.^77^ Augmenting inhibitory processes exerts a therapeutic effect in epilepsy by counteracting cortical hyperexcitability and thereby reducing the likelihood of hypersynchronous epileptic discharges.^78^

We also found effects regarding connectivity features. Alpha connectivity was diminished in the AED (Na) group (Fig. 11C, right, Fig. 10N), while theta (Fig. 10B–H, Fig. 11C, left) coherence was predominantly increased after AED treatment. The results of previous studies are inconsistent. Kim et al.^79^ found no significant differences in resting-state EEG coherence after oxcarbazepine treatment. On the other hand, Clemens et al.^80^ showed that valproic acid normalises theta and alpha connectivity in patients with epilepsy.

## Discussion

We investigated the pharmaco-EEG effects of several drug classes, using PCA as a dimensionality reduction technique to integrate results from numerous EEG features.

**Fig. 12:**
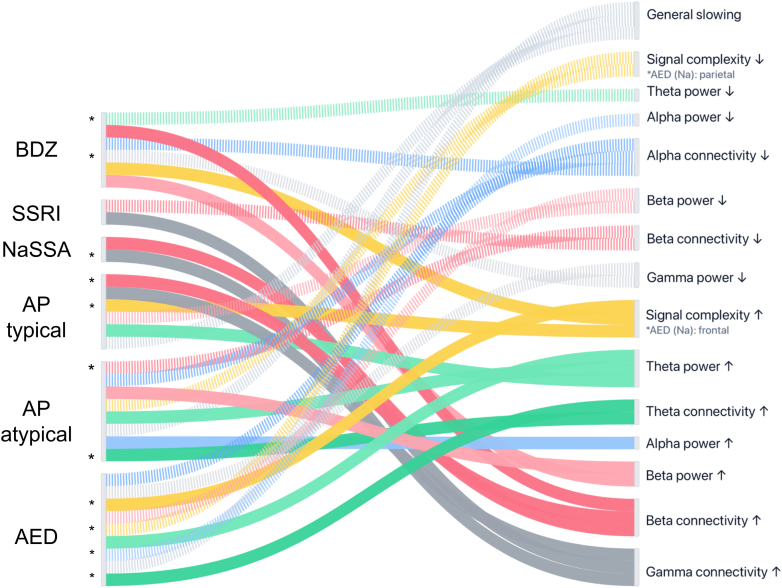
Summary of significant resting-state EEG effects of different drug classes. Asterisk marks effects that were not reported previously.

We observed significant effects among five drug classes: benzodiazepines correlated with increased signal complexity, increased beta and reduced theta, alpha and gamma power; SSRIs were associated with altered coherence by enhancing it in gamma and reducing it in beta frequency; and NaSSA with increased gamma coherence. Patients treated with antipsychotics or anticonvulsants exhibited general EEG rhythm slowing, elevated slow-wave power, and altered signal complexity. Fig. 12 presents a summary of the effects found by our analysis for the five drug classes described above. We did not find any significant differences in PCA components for other drug classes; however, for SARI and SNRI, there were differences in single features (1983 and 1548 significant features, respectively). We will not discuss them here, but we encourage the reader to check the effects in our interactive database at https://brainwavesrx.nencki.edu.pl/.

While the observed effects largely accord with prior literature, the scale of our study provides substantially greater statistical power, allowing us to detect subtler and more distributed effects and identify patterns that are difficult to resolve using smaller samples. The PCs with the strongest effect sizes were significant in both the one-vs-rest and medication-vs-drug-naïve contrasts (see Tables S1–S6), suggesting that these effects are robust across different reference groups and may reflect medication-specific EEG patterns.

Notably, statistically significant effects in coherence features emerged across all drug classes, whereas the existing literature focuses mostly on the spectral effects of psychotropic drugs. Indeed, coherence measures track communication between brain regions,^81^^,^^82^ which is primarily what neuromodulators (the targets of the majority of psychotropic drugs) alter.^83^ Coherence may then be more sensitive to neuropsychiatric medicines' effects, as suggested also in the paper by Ford et al.^84^ Due to this, coherence as a potential drug administration biomarker should be studied more. On the other hand, spectral and nonlinear features were also important for all drug classes except SSRI + SMS and NaSSA, illustrating that different categories of EEG metrics capture distinct aspects of the EEG differences associated with these medication groups. Different feature types complement each other, and multi-feature analyses can support a broader characterisation of EEG patterns observed across patients taking different psychotropic drugs.

Several PCs were significantly differentiating more than one group of medicines. For example, PC1 (Fig. S2, top), gathering beta and gamma connectivity features, was significant for SSRI, NaSSA, and atypical antipsychotics. Analysis of the individual features contributing to this PC showed that all three drug classes were associated with increased gamma coherence, whereas beta coherence was decreased in the SSRI group and increased in the NaSSA and atypical antipsychotic groups. A common element of the mechanisms of action of these drugs is modulation of the serotonergic system. This observation suggests that gamma coherence may be related to serotonergic activity — a connection also proposed by Lee et al.^85^ Indeed, studies show that antagonising serotonin receptors pharmacologically causes increases in EEG gamma power,^86^ while serotonin injection suppresses gamma oscillations,^87^ which may provide one possible interpretive context for the present findings.

Another component, PC6 (Fig. S2, middle), which encompasses decreased signal complexity, increased theta/low-alpha coherence and power, and decreased beta power, was significant for benzodiazepines, anticonvulsants (Na-channel blocking), and atypical antipsychotics. Both antipsychotic and anticonvulsant drugs have been described in the literature as being associated with reduced cortical excitability and stabilised neural network activity (anticonvulsants directly by enhancing GABAergic inhibition or reducing glutamatergic excitation,^77^ while antipsychotics indirectly through dopamine D2 antagonism or serotonin receptor modulation).^3^ In prior literature, such pharmacological effects have been associated with EEG patterns including lower signal complexity, higher low-frequency power, and lower high-frequency power, reflecting a move toward more regularised and less hyperexcitable brain states.^75^^,^^76^^,^^78^ For benzodiazepines, this effect was in the opposite direction, indicating higher signal complexity and beta power in the BDZ group. Prior work shows that BDZs enhance inhibitory GABA receptors, which results in larger/longer inhibitory postsynaptic currents,^88^ especially at perisomatic synapses from parvalbumin-positive interneurons to pyramidal cells, which are rich in benzodiazepine-sensitive GABA_A_ receptors.^89^^,^^90^ This mechanism has been proposed to contribute to increased synchrony of the pyramidal neurons, which is visible as increased beta power in EEG.^91^ In contrast, atypical antipsychotics have been reported to antagonise serotonin receptors, and this action leads to decreased excitability of layer V pyramidal neurons,^92^ which has been suggested to be the central drivers of beta rhythms. Similarly, AEDs have been associated with reduced pyramidal-interneuron excitability, as they block voltage-gated sodium or calcium channels.^93^

As excitability drops, cortical networks shift toward more stable membrane potentials and stronger low-frequency synchronisation,^94^ which may be relevant to theta/low-alpha coherence and power changes contributing to PC6 (Fig. S2E and F). Indeed, the fact that these features were gathered into one PC probably indicates that they covary systematically across patients, so we hypothesise they may result from the same neural mechanisms.

Another component significant for BDZ, AED (Na), and typical APs was PC7 (Fig. S2, bottom), which primarily captured increased theta/low-alpha power and coherence. The overall slowing of EEG activity and the occurrence of lower-frequency oscillations have often been linked to reduced arousal levels.^95^^,^^96^ As noted above, AEDs and APs have been associated with reduced cortical excitability, whereas BDZs are commonly associated with sedative effects. Thus, the convergence of these three classes on PC7 may be consistent with prior reports linking them to lower arousal and/or altered excitability, together with a shift toward slower, more synchronised dynamics.

Generally, an interesting interaction was observed between BDZ and antipsychotic drugs. BDZ showed effects in the opposite direction to atypical APs in several PCs, including PC6 (Fig. S2, middle), PC18 (Fig. S3, middle), and PC36 (Fig. S3, bottom), but in the same direction as typical APs in PC7 (Fig. S2, bottom) and PC11 (Fig. S3, top). These PCs were driven largely by complexity features and beta-band power. BDZ and typical APs were associated with increased beta power and greater signal complexity, whereas atypical APs showed the opposite pattern. Benzodiazepines, through modulation of GABA_A_ receptors, have been linked with increased fast inhibitory rhythmic activity and nonlinear EEG coupling.^52^^,^^97^ Typical antipsychotics may produce a partially similar EEG profile in some contexts, as haloperidol has been reported to increase beta-band power and prolong EEG microstate duration.^65^ By contrast, atypical antipsychotics–particularly clozapine and olanzapine–were more commonly associated with shifting EEG activity toward slower delta/theta rhythms and reduced beta activity, a pattern that may be accompanied by lower apparent signal complexity.^98^ A study examining EEG changes following a switch from typical to atypical antipsychotic treatment reported increased theta and low-alpha power together with decreased beta power,^99^ consistent with the contrasting patterns associated with the two antipsychotic classes in our data.

Taken together, our results suggest that jointly modelling spectral, coherence, and nonlinear features, and gathering them into principal components for a more integrated view, enables a robust characterisation of the complex and multidimensional EEG patterns associated with different psychiatric medications. Our study enhances statistical power while providing a broader and mechanistically informative picture of medication-associated EEG differences, thereby offering a useful framework for future biomarker-oriented research and for further investigation of the neurophysiological correlates of psychotropic medication use.

Our study was not free from limitations, though. Firstly, there was a discrepancy in group sizes, resulting in unequal statistical power between drug classes. Notably, the drug classes for which no significant PCA effects were detected had smaller sample sizes than those showing significant effects. Thus, we cannot exclude that classes with larger samples simply yielded more significant findings due to greater statistical power rather than stronger pharmaco-EEG effects. However, it is important to note that we employed the effect-size statistic that is relatively robust to small-sample bias.^51^

An additional limitation is the lack of analysis of multidrug therapy. Many patients in the dataset were treated with more than one psychotropic medication, yet potential drug interactions were not examined. We plan to explore this in a further study. At the same time, we believe that the effects identified here are not driven primarily by co-therapy, as in the one-vs-rest design, we balanced the comparison groups with respect to the distribution of medications other than the drug class of interest (see Figs. S4–S9). Moreover, most of the effects were confirmed by the sensitivity analysis, where we excluded patients treated with more than one drug class (see Supplementary Tables S1–S6). Still, some findings should be interpreted with caution. In particular, principal components shared by benzodiazepines and antipsychotics may partly reflect the frequent co-administration of these two drug classes, seen in Fig. S12B.

As this was a retrospective study, we had no control over the timing of EEG recordings, and patients were at varying stages of pharmacotherapy at the time of assessment. The variability in this aspect of the data may be one of the reasons why our reported effects were predominantly of small size. Nevertheless, we replicated many previously reported findings based on precisely timed recordings, including several effects emerging just after drug administration. This consistency suggests that the pharmaco-EEG patterns we observed are stable during long-term treatment.

In addition, information regarding medication dosage could not be incorporated into the analysis due to substantial amounts of missing data. Similarly, we were unable to distinguish between treatment responders and non-responders, as data on therapeutic outcomes were unavailable. What is more, we had no information about other clinical variables, such as the severity of the condition, the disease stage, or the symptomatic status. It is acknowledged that factors such as dosage, treatment resistance, and disease stage may influence pharmacotherapy-induced EEG alterations, and the inability to account for these variables constitutes a limitation of the present study, as the heterogeneity in our data, together with the cross-sectional design, makes it difficult to distinguish pharmacological effects from pre-existing EEG characteristics of the patients. At the same time, we believe that the very large sample size and the naturalistic clinical character of the dataset still provide valuable information on medication-associated EEG patterns at the population level.

Despite these constraints, the study offers substantial value. Its large sample size, multi-feature EEG analysis, and integration of heterogeneous real-world clinical data allow us to capture drug-related neural signatures that may be less apparent in smaller, controlled experiments. Our findings contribute important large-scale evidence on the electrophysiological signatures of major psychotropic drug classes and highlight promising neural biomarkers for future research. In particular, the EEG features identified here as differing between patients treated with specific psychotropic drug classes and other patients may help define candidate markers for subsequent treatment-response analyses.

## Contributors

Magdalena Szponar–data analysis, data curation, methodology, literature search, visualisation, writing—original draft.

Patrycja Dzianok–software, online database management, visualisation, writing—review & editing.

Bartłomiej Gmaj–data collection, data curation, writing—review & editing.

Wojciech Jernajczyk–data collection, data curation, writing—review & editing.

Jan Kamiński–conceptualisation, funding acquisition, methodology, project administration, supervision, writing—review & editing.

Magdalena Szponar, Patrycja Dzianok and Jan Kamiński accessed and verified the data used in the manuscript (calculated EEG features). Magdalena Szponar, Wojciech Jernajczyk and Bartłomiej Gmaj had access to and verified the raw data (EEG recordings).

All authors have read and approved the final version of the manuscript.

## Data sharing statement

The results from all comparisons between all drug classes are available on the interactive website, at https://brainwavesrx.nencki.edu.pl/.

The code used for data preprocessing and statistical analysis is available on GitHub at https://github.com/labianca/EEG-psychotropic-medications.

## Declaration of interests

The authors declare no conflicts of interest.
