# Supplementary material for: Brainwaves under medication: revealing class-specific neural signatures of psychotropic medication from 24,000 EEGs
Source: eBioMedicine. 2026 Jul 9;130:106375. doi: 10.1016/j.ebiom.2026.106375 (PMC13380497; doi:10.1016/j.ebiom.2026.106375)
Supplement: Supplementary Figures and Tables [file mmc1.pdf]

Brainwaves Under Medication - Supplementary material

Contents

Effect sizes and the most contributing features for PCA components that differ significantly between medicines in one-vs-rest comparison (Tables S1-S6)……….…… 2

Top 10 PCs (Figure S1)………………………………………………………………….. 8

The PCs that show shared effects between drug classes (Figures S2-S3)……………. 10

Age, sex, and diagnosis distributions of matched groups (Figures S4-S9).....................12

Graphical summary of the applied methodology (Figure S10) ..…………………………18

Additional characteristics of the patients' sample…………………………………...…19

Disorder distribution among the patients (Figure S11)…………………………………19

Multidrug therapy (Figure S12)...19

Recording time and hospital site in comparison groups (Figures S13-S18)……….…… 20

Preprocessing (Figures S19-S20)..………………………………………………………….. 26

Table S7. Preprocessing pipeline pseudocode……………..………………………..26

Effect sizes and the most contributing features for PCA components that differ significantly between medicines in one-vs-rest comparison.

Table S1. Significant PCs for Benzodiazepines (ordered by OvR effect size). The column N match denotes how many out of 10 matches the component was significant in. Effect sizes and CI's are averaged from ten matchings.

PC	Var explain-ed (%)	Effect vs other medicines			Single-class effect vs other medicines			Effect vs drug naive		
		p_{adj}	Hedges' g [95% CI]	N match	p_{adj}	Hedges' g [95% CI]	N match	p_{adj}	Hedges' g [95% CI]	N match
3	3.76	**< 0.001**	0.34 [0.28,0.39]	10	**< 0.001**	0.36 [0.22,0.5]	9	**< 0.001**	0.38 [0.33,0.44]	10
	increased **beta power**, decreased gamma power; increased temporo-parietal signal complexity; decreased alpha coherence between frontal and occipital areas									
11	1.22	**< 0.001**	0.29 [0.23,0.34]	10	n.s.	0.25 [0.11,0.39]	3	**< 0.001**	0.40 [0.34,0.45]	10
	increased **frontal** signal **complexity** and **beta\low-gamma power**; increased low-alpha and beta connectivity									
27	0.55	**< 0.001**	0.19 [0.13,0.24]	10	n.s.	0.25 [0.11,0.39]	0	**< 0.001**	0.20 [0.14,0.25]	10
	**increased** low-alpha and gamma connectivity; **decreased** theta, high-alpha and beta **connectivity**									
6	2.05	**< 0.001**	0.16 [0.11,0.22]	10	n.s.	0.28 [0.15,0.42]	3	n.s.	0.08 [0.02,0.13]	0
	increased whole-brain signal **complexity**; decreased theta-low/alpha and increased high-alpha **connectivity**, both increased and decreased beta connectivity; **elevated beta** and **reduced low-alpha powe**r in occipital regions									
37	0.42	**< 0.001**	0.15 [0.1,0.21]	10	n.s.	0.15 [0.01,0.29]	0	**< 0.001**	0.23 [0.17,0.28]	10
	increased central signal **complexity**; increased **beta power** and decreased **theta power**; connectivity features at a wide frequency range, both increased and decreased									
18	0.76	**< 0.001**	0.15 [0.1,0.21]	10	n.s.	0.15 [0.01,0.28]	0	**< 0.001**	0.18 [0.13,0.24]	10
	increased signal **complexity;** increased **beta power**; **connectivity** features at a wide frequency range, both increased and decreased									
40	0.39	**< 0.001**	0.15 [0.1,0.21]	10	n.s.	0.14 [0.01,0.27]	0	**< 0.001**	0.18 [0.12,0.23]	10
	increased **beta power** and decreased **gamma power; connectivity** features at a wide frequency range, both increased and decreased; decreased occipital signal complexity									
38	0.41	**< 0.001**	0.15 [0.1, 0.21]	10	n.s.	0.24 [0.1,0.37]	1	**0.002**	0.13 [0.08,0.18]	10
	increased **alpha** and **beta connectivity**; increased **beta power;** decreased temporal signal complexity									

PC	Var explain-ed (%)	Effect vs other medicines			Single-class effect vs other medicines			Effect vs drug naive		
		p_{adj}	Hedges' g [95% CI]	N match	p_{adj}	Hedges' g [95% CI]	N match	p_{adj}	Hedges' g [95% CI]	N match
31	0.49	**< 0.001**	0.15 [0.09,0.2]	10	n.s.	0.11 [0.01,0.29]	0	**< 0.001**	0.15 [0.1,0.21]	10
	**connectivity** features at a wide frequency range, both increased and decreased, increased **beta power**									
7	1.72	**< 0.001**	0.15 [0.09,0.2]	10	n.s.	0.14 [0.07,0.21]	0	**< 0.001**	0.32 [0.27,0.38]	10
	increased **theta/low-alpha connectivity** and power, increased temporo-occipital complexity, decreased parietal complexity									
26	0.59	**< 0.001**	0.14 [0.09,0.2]	9	n.s.	0.24 [0.14,0.42]	4	**< 0.001**	0.16 [0.1,0.21]	10
	**connectivity** features at a wide frequency range, both increased and decreased, increased frontal **beta power**									
41	0.38	**0.001**	0.14 [0.08,0.19]	10	n.s.	0.17 [0.01,0.27]	0	**< 0.001**	0.17 [0.12,0.22]	10
	increased frontal low-beta and gamma **power**, decreased theta and high-beta power; decreased **alpha connectivity**; increased frontal signal complexity									
16	0.86	**0.005**	0.13 [0.07,0.18]	7	n.s.	0.18 [0.01,0.29]	0	**< 0.001**	0.20 [0.15,0.25]	10
	increased low-**alpha connectivity and occipital power**, decreased high alpha connectivity									
36	0.43	**0.016**	0.12 [0.07,0.18]	7	n.s.	0.18 [0.01,0.29]	0	**0.002**	0.13 [0.08,0.19]	10
	increased **beta power**, increased **low-alpha connectivity**, increased **signal complexity**									
33	0.46	**0.010**	-0.12 [0.07,0.18]	8	n.s.	0.11 [0.04,0.24]	0	**< 0.001**	0.14 [0.09,0.2]	10
	**connectivity** features at a wide frequency range, both increased and decreased, decreased **alpha frontal power**									

sum: 14.51

Table S2. Significant PC for SSRIs.

PC	Var explained (%)	Effect vs other medicines			Single-class effect vs other medicines			Effect vs drug naive		
		p_{adj}	Hedges' g [95% CI]	N match	p_{adj}	Hedges' g [95% CI]	N match	p_{adj}	Hedges' g [95% CI]	N match
10	1.39	**< 0.001**	0.18 [0.12,0.23]	10	**< 0.001**	0.32 [0.22,0.42]	10	**< 0.001**	0.22 [0.16,0.27]	10
	increased **theta/low-alpha** and **gamma** connectivity, mostly decreased **beta connectivity**									
1	6.20	**< 0.001**	0.17 [0.12,0.23]	10	**< 0.001**	0.26 [0.16,0.36]	10	**< 0.001**	0.16 [0.1,0.21]	10
	decreased **beta** connectivity, increased **gamma connectivity**									

sum: 7.59

Table S3. Significant PC for NaSSA.

PC	Var explained (%)	Effect vs other medicines			Single-class effect vs other medicines			Effect vs drug naive		
		p_{adj}	Hedges' g	N match	p_{adj}	Hedges' g	N match	p_{adj}	Hedges' g	N match
1	6.20	**< 0.001**	0.38 [0.30,0.47]	10	**0.010**	0.47 [0.29,0.65]	10	**< 0.001**	0.31 [0.23,0,40]	10
	decreased **beta** connectivity, increased **gamma connectivity**									

sum: 6.20

Table S4. Significant PCs for atypical antipsychotics (ordered by OvR effect size).

PC	Var explain-ed (%)	Effect vs other medicines			Single-class effect vs other medicines			Effect vs drug naive		
		p_{adj}	Hedges' g	N match	p_{adj}	Hedges' g	N match	p_{adj}	Hedges' g	N match
1	6.20	**< 0.001**	0.27 [0.24,0.31]	10	**< 0.001**	0.32 [0.26,0.38]	10	**< 0.001**	0.19 [0.14,0.24]	10
	increased **beta** connectivity, decreased **gamma connectivity**									
9	1.51	**< 0.001**	0.18 [0.15,0.22]	10	**< 0.001**	0.21 [0.15,0.27]	10	**< 0.001**	0.17 [0.12,0.22]	10
	**decreased signal complexity**; increased **theta power**; decreased **alpha** and increased **gamma connectivity**									
13	1.04	**< 0.001**	0.18 [0.14,0.22]	10	**< 0.001**	0.23 [0.17,0.29]	10	**< 0.001**	0.19 [0.14,0.24]	10
	mostly decreased **beta connectivity**; decreased frontal signal **complexity**; increased low-alpha frontal power and connectivity									
4	3.51	**< 0.001**	0.13 [0.1,0.17]	10	n.s.	0.10 [0.04,0.16]	0	n.s.	0.10 [0.05,0.15]	0
	decreased frontal and occipital signal complexity; increased **alpha** and **beta connectivity**									
36	0.43	**< 0.001**	0.13 [0.09,0.16]	10	**0.016**	0.13 [0.07,0.18]	8	n.s.	0.04 [0.01,0.09]	0
	decreased **theta/low-alpha connectivity**; decreased **beta** power and **signal complexity** in the **fronto-central area**									
29	0.52	**< 0.001**	0.11 [0.08,0.15]	10	n.s.	0.05 [0.0,0.11]	0	n.s.	0.09 [0.04,0.14]	0
	increased **frontal signal complexity**; decreased **theta/low-alpha connectivity** and power; increased **high-alpha** power and decreased **beta power**									
6	2.05	**< 0.001**	0.11 [0.07,0.15]	10	n.s.	0.11 [0.06,0.17]	3	**< 0.001**	0.14 [0.09,0.19]	10
	decreased whole-brain signal **complexity**; increased theta-low/alpha and decreased high-alpha **connectivity**, both increased and decreased beta connectivity; **elevated low-alpha** and **reduced beta powe**r in occipital regions									
8	1.60	**< 0.001**	0.10 [0.07,0.14]	10	n.s.	0.15 [0.06,0.17]	2	**0.006**	0.12 [0.07,0.17]	9
	**gamma connectivity**, both increased and decreased									
57	0.26	**< 0.001**	0.10 [0.06,0.14]	10	n.s	0.07 [0.02,0.13]	0	**0.054**	0.11 [0.06,0.16]	0
	mostly increased low-frequency **connectivity**, decreased low-beta while increased high-beta **connectivity**; decreased **theta/low-alpha** power, increased **low-beta power**									
10	1.39	**0.001**	0.09 [0.06,0.13]	10	**< 0.001**	0.16 [0.11,0.22]	10	**0.004**	0.12 [0.07,0.17]	7
	increased **theta/low-alpha** and **gamma** connectivity, mostly decreased **beta connectivity**									

PC	Var explained (%)	Effect vs other medicines			Single-class effect vs other medicines			Effect vs drug naive		
		p_{adj}	Hedges' g	N match	p_{adj}	Hedges' g	N match	p_{adj}	Hedges' g	N match
166	0.08	**0.001**	0.09 [0.06,0.13]	10	n.s.	0.06 [0.01,0.12]	0	**0.021**	0.11 [0.06,0.16]	6
	**connectivity** features at a wide frequency range, both increased and decreased, increased alpha and low-gamma **power** in temporal areas; decreased fronto-central signal **complexity**									
18	0.76	**0.002**	0.09 [0.05,0.13]	9	n.s.	0.12 [0.06,0.17]	3	n.s.	0.01 [0.04,0.06]	0
	decreased signal **complexity;** decreased **beta power**; **connectivity** features at a wide frequency range, both increased and decreased									
93	0.15	**0.003**	0.09 [0.05,0.12]	9	n.s.	0.08 [0.02,0.13]	0	**0.002**	0.12 [0.07,0.17]	10
	**connectivity** features at a wide frequency range, both increased and decreased, decreased **low-beta power**									
294	0.04	**0.004**	0.09 [0.05,0.12]	9	n.s.	0.07 [0.01,0.13]	0	n.s.	0.08 [0.03,0.13]	0
	decreased **frontal** signal **complexity; connectivity** features at a wide frequency range, both increased and decreased, power features at a wide frequency range, both increased and decreased									
274	0.04	**0.013**	0.08 [0.05,0.12]	9	n.s.	0.08 [0.03,0.14]	0	n.s.	0.07 [0.02,0.12]	0
	decreased **frontal** signal **complexity; connectivity** features at a wide frequency range, both increased and decreased									

sum: 19.56

Table S5. Significant PCs for typical antipsychotics (ordered by OvR effect size).

PC	Var explained (%)	Effect vs other medicines			Single-class effect vs other medicines			Effect vs drug naive		
		p_{adj}	Hedges' g	N match	p_{adj}	Hedges' g	N match	p_{adj}	Hedges' g	N match
11	1.22	**< 0.001**	0.17 [0.11,0.22]	10	**0.005**	0.25 [0.15,0.35]	9	**< 0.001**	0.32 [0.26,0.37]	10
	increased **frontal** signal **complexity** and **beta\low-gamma power**; increased low-alpha and beta connectivity									
7	1.72	**0.001**	0.14 [0.09,0.19]	10	n.s.	0.10 [0.09,0.11]	0	**< 0.001**	0.36 [0.3,0.41]	10
	increased **theta/low-alpha connectivity** and power, increased temporo-occipital complexity, decreased parietal complexity									

sum: 2.95

Table S6. Significant PCs for sodium channel blocking anticonvulsants (ordered by OvR effect size).

PC	Var explain-ed (%)	Effect vs other medicines			Single-class effect vs other medicines			Effect vs drug naive		
		p_{adj}	Hedges' g	N match	p_{adj}	Hedges' g	N match	p_{adj}	Hedges' g	N match
7	1.72	**< 0.001**	0.21 [0.17,0.26]	10	**0.037**	0.20 [0.11,0.29]	6	**< 0.001**	0.35 [0.3,0.4]	10
	increased **theta/low-alpha connectivity** and power, increased temporo-occipital complexity, decreased parietal complexity									
6	2.05	**< 0.001**	0.18 [0.14,0.23]	10	**< 0.001**	0.23 [0.14,0.32]	8	**< 0.001**	0.22 [0.17,0.27]	10
	decreased whole-brain signal complexity; increased theta-low/alpha and decreased high-alpha connectivity, both increased and decreased beta connectivity; elevated low-alpha and reduced beta power in occipital regions									
3	3.76	**< 0.001**	0.13 [0.08,0.17]	10	n.s.	0.06 [0.03,0.15]	0	**< 0.001**	0.19 [0.14,0.24]	10
	increased **beta power**, decreased gamma power; increased temporo-parietal signal complexity; decreased alpha coherence between frontal and occipital areas									
17	0.83	**< 0.001**	0.13 [0.08,0.17]	10	n.s.	0.14 [0.04,0.23]	0	**< 0.001**	0.15 [0.1,0.2]	10
	increased **theta/low-alpha** and **beta connectivity**									
20	0.72	**0.001**	0.12 [0.08,0.17]	10	n.s.	0.12 [0.03,0.22]	0	n.s.	0.10 [0.05,0.15]	0
	decreased occipital signal **complexity**, but increased complexity in other areas; decreased **theta** and **alpha connectivity**									
19	0.74	**0.004**	0.11 [0.06,0.15]	7	n.s.	0.08 [0.02,0.17]	0	**< 0.001**	0.13 [0.08,0.18]	10
	increased signal **complexity**; increased **low-beta occipital power**; increased low-alpha connectivity									
106	0.13	**0.036**	0.10 [0.05,0.14]	7	n.s.	0.09 [0.03,0.16]	0	**< 0.001**	0.14 [0.09,0.19]	10
	increased theta and alpha **connectivity**; decreased **alpha power**; decreased occipital signal **complexity**									

sum: 9.97

Top 10 PCs

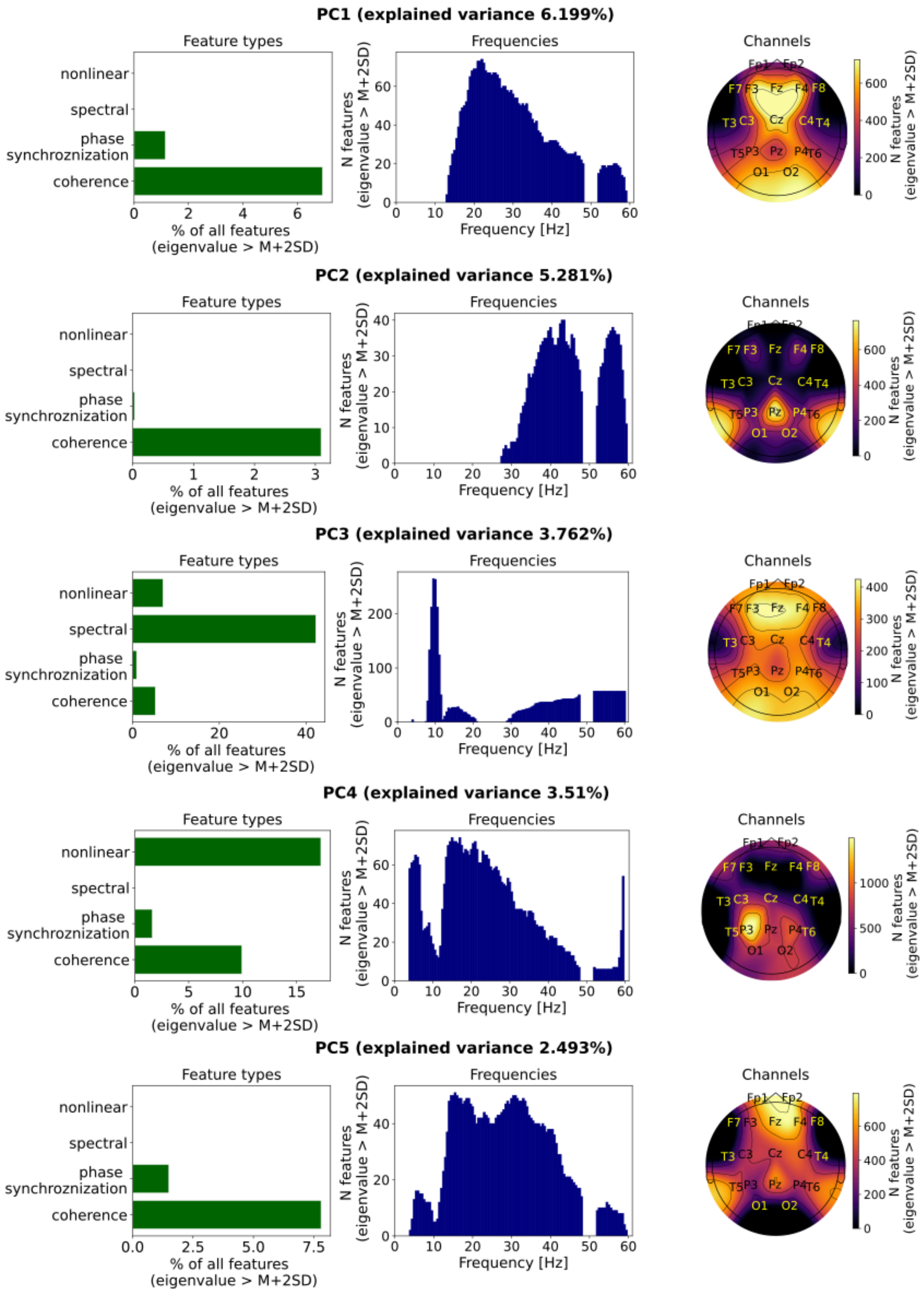

Figure S1. Features contributing the most to the top 10 principal components that explain the most variance, depending on different domains: left, based on the type of analysis; middle, for different spectral contributions; and right, for scalp localization.

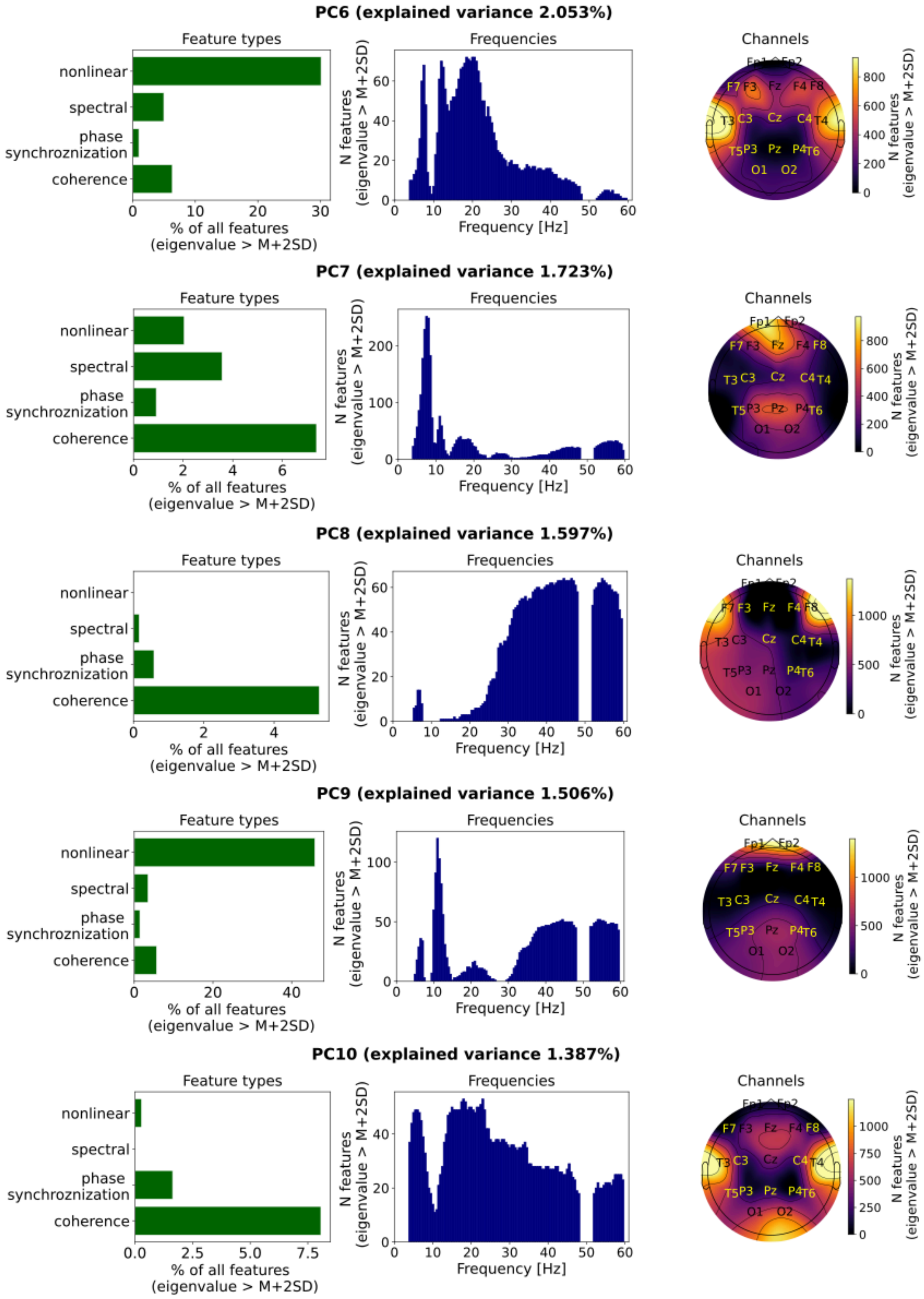

Figure S1 (continued).

The PCs that show shared effects between drug classes

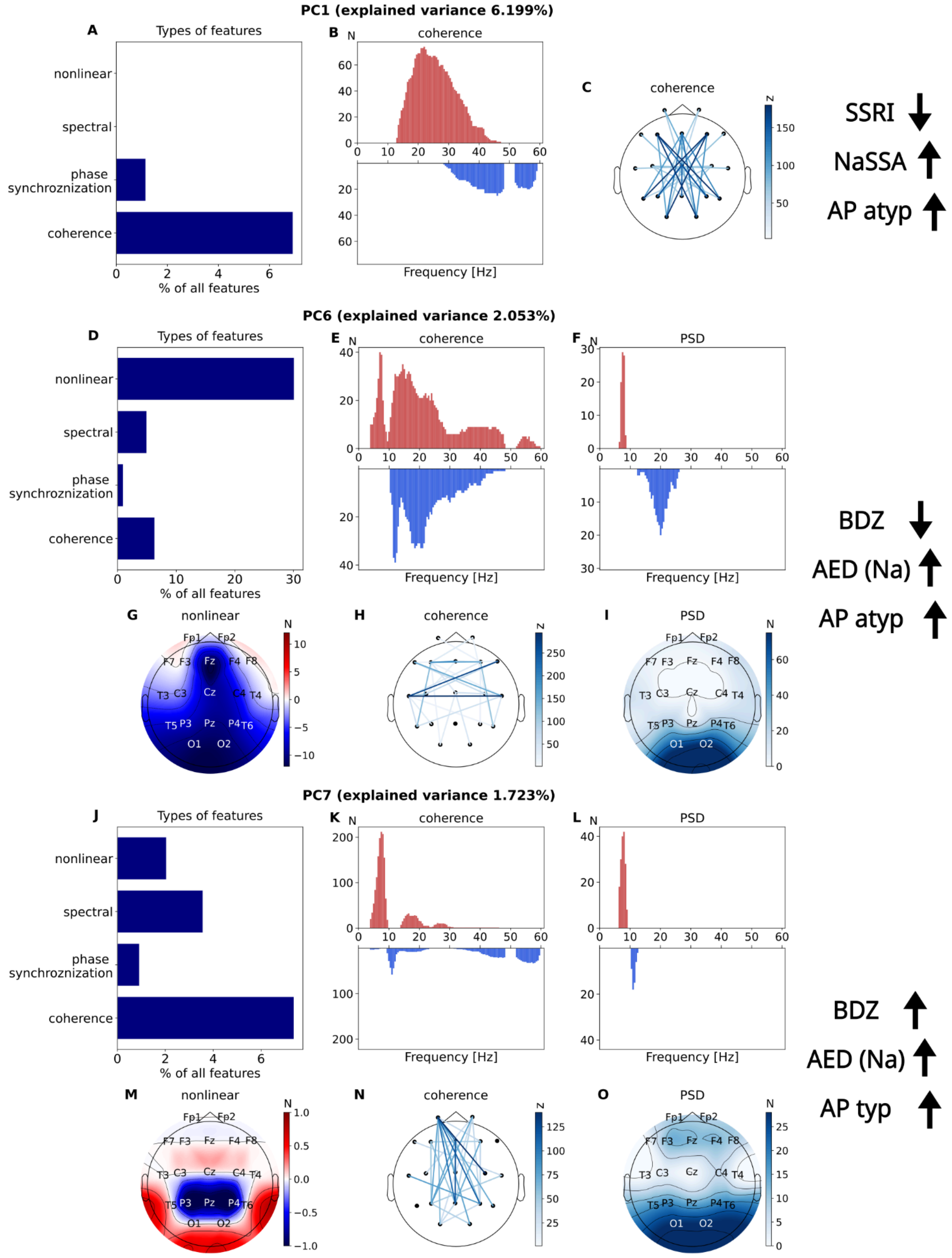

Figure S2. PCA components that show shared effects between three types of drugs. PC1 (top) was significant for NaSSA, AP atypical, and SSRI, with SSRI showing the opposite effect to the other drugs. PC6 was significant for BDZ, AP atypical, and AED (Na), with BDZ showing an opposite effect than the other classes. PC7 was significant for BDZ, AP typical, and AED(Na), with all drugs showing the same effects. **A, D, J.** The percentage of high eigenvalues from each type of EEG features. **B, E, F, K,** and **L** represent the number of features as a function of frequency, separately for connectivity measures (**B, E, K**) and PSD (**F, L**). The frequencies that have positive loadings into the given PC are shown in red, while those with negative loadings are shown in blue. **C, G-I,** and **M-O** show the number of features as a function of localization, separately for connectivity measures (**C, H, N**), PSD (**I, O**), and nonlinear features (**G, M**). For nonlinear measures, the electrodess with positive loadings into the given PC are shown in red, while those with negative loadings are shown in blue. For PSD, an absolute value is shown. For connectivity, 30 connections with the highest number of contributing features are shown.

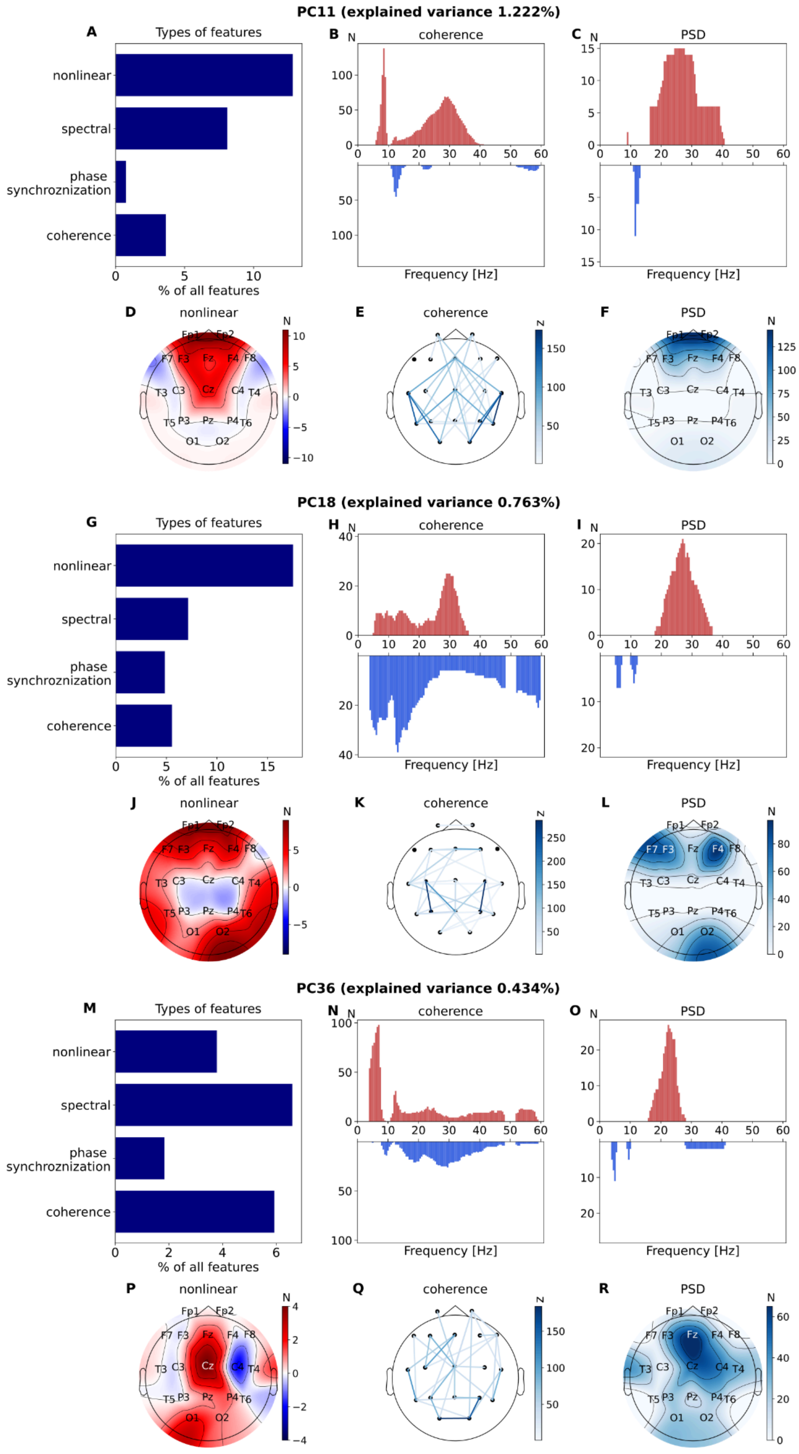

Figure S3. PCA components that show shared effects between antipsychotics and benzodiazepines. BDZ showed opposite effects to atypical APs in PCs 18 and 36, but they showed effects in the same direction as typical APs in PC11. **A, G, M.** The percentage of high eigenvalues from each type of EEG features. **B, C, H, I, N,** and **O** represent the number of features as a function of frequency, separately for connectivity measures (**B, H, N**) and PSD (**C, I, O**). The frequencies that have positive loadings into the given PC are shown in red, while those with negative loadings are shown in blue. **D-F, J-L,** and **P-R** show the number of features as a function of localization, separately for connectivity measures (**E, K, Q**), PSD (**F, L, R**), and nonlinear features (**D, J, P**). For nonlinear measures, the electrodes with positive loadings into the given PC are shown in red, while those with negative loadings are shown in blue. For PSD, an absolute value is shown. For connectivity, 30 connections with the highest number of contributing features are shown.

Age, sex, and diagnosis distributions of matched groups

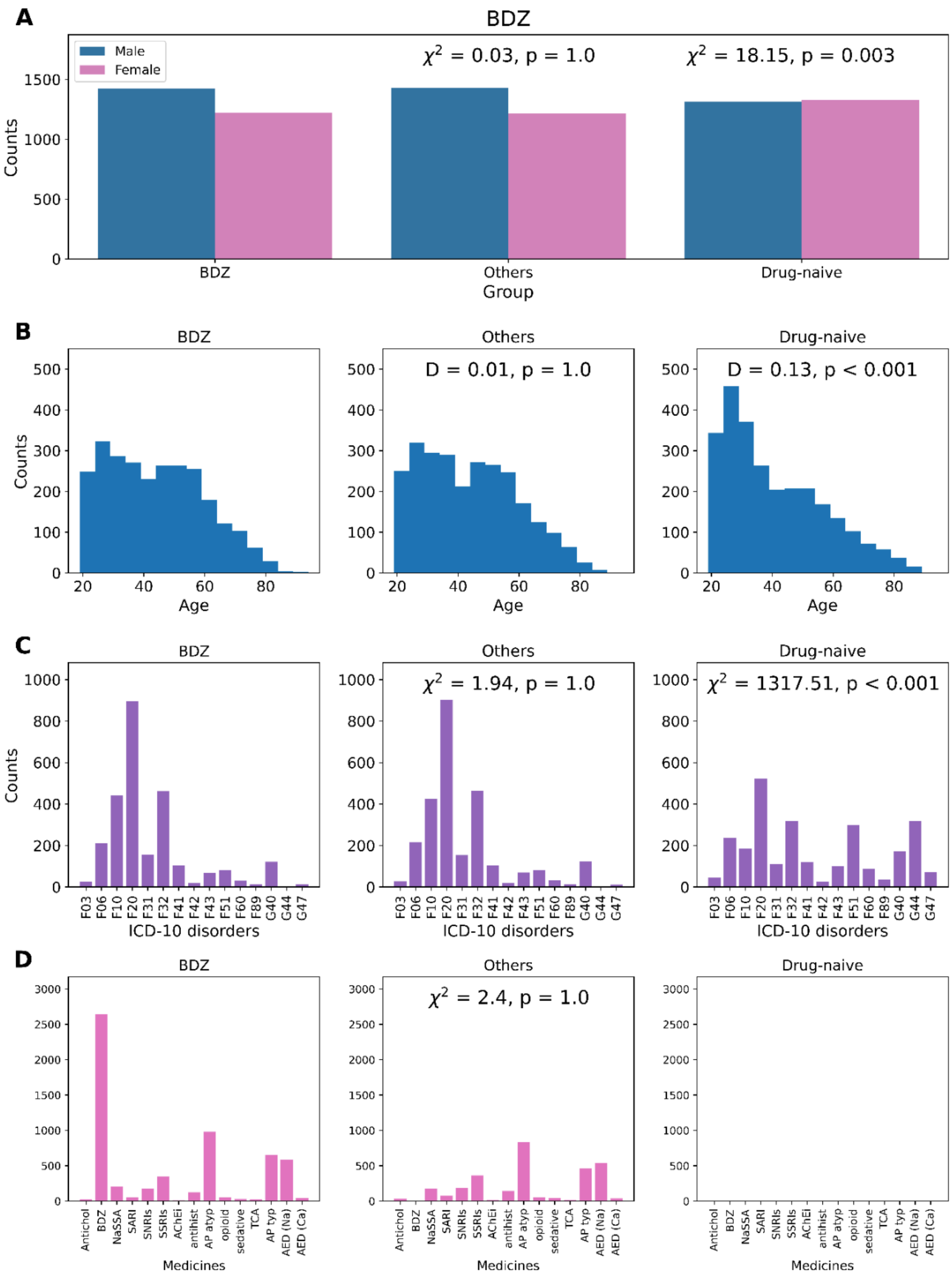

Figure S4. Age, sex, diagnosis and medication distributions for Benzodiazepine users and matched groups. Statistics (Kolmogorov–Smirnov D for age and X^2 for other graphs) and p-values show whether the difference between the distributions in the medicine group and other groups was significant. **A.** Sex distribution for BDZ (left), other drugs (middle), and drug-naive (right) groups. **B.** Age distribution (in 5-year bins) for BDZ (left), other drugs (middle), and drug-naive (right) groups. **C.** Diagnosis (in ICD-10 codes) distribution for BDZ (left), other drugs (middle), and drug-naive (right) groups. **D**. Medicines taken count in BDZ (left), other drugs (middle), and drug-naive (right) groups. For this graph, distribution comparison statistics are calculated only for medicines other than BDZ in the OvR comparison.

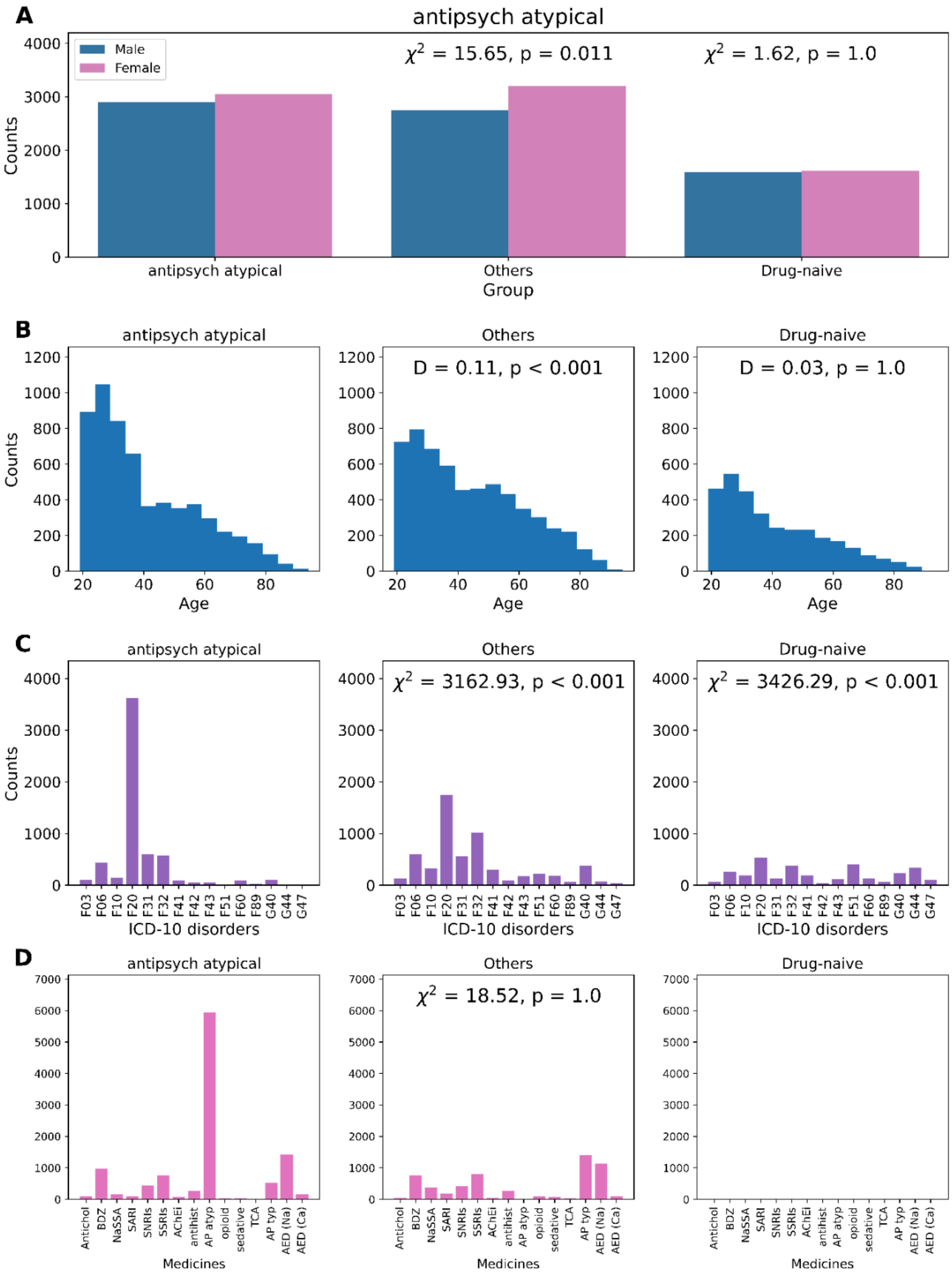

Figure S5. Age, sex, and diagnosis distributions for atypical antipsychotic users and matched groups. Statistics (Kolmogorov–Smirnov D for age and X^2 for other graphs) and p-values show whether the difference between the distributions in the medicine group and other groups was significant (Bonferroni-Holm corrected). **A.** Sex distribution for atypical APs (left), other drugs (middle), and drug-naive (right) groups. **B.** Age distribution (in 5-year bins) for atypical APs (left), other drugs (middle), and drug-naive (right) groups. **C.** Diagnosis (in ICD-10 codes) distribution for atypical APs (left), other drugs (middle), and drug-naive (right) groups. **D**. Medicines taken count in atypical APs (left), other drugs (middle), and drug-naive (right) groups. For this graph, distribution comparison statistics are calculated only for medicines other than atypical APs in OvR comparison.

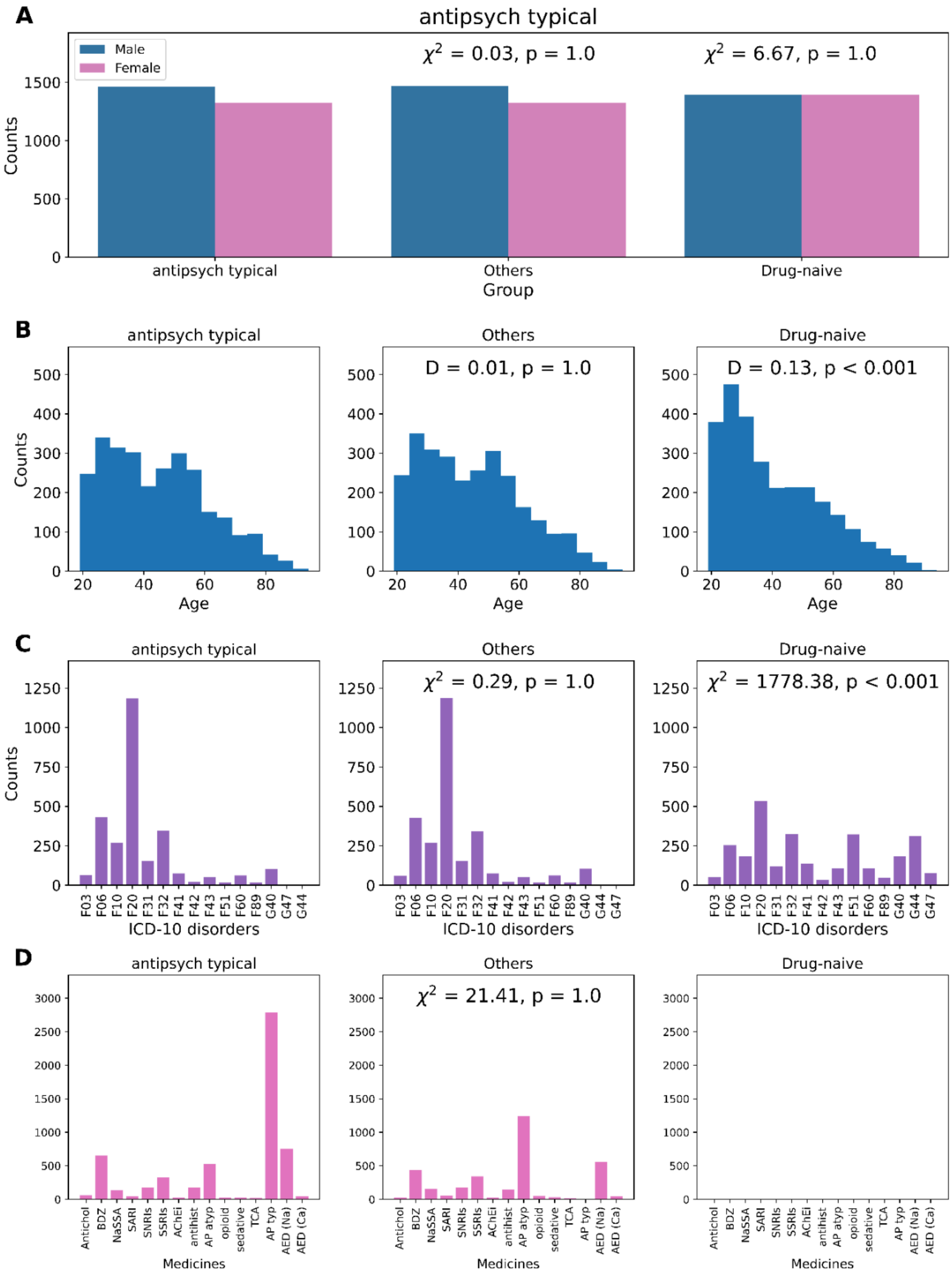

Figure S6. Age, sex, and diagnosis distributions for typical antipsychotic users and matched groups. Statistics (Kolmogorov–Smirnov D for age and X^2 for other graphs) and p-values show whether the difference between the distributions in the medicine group and other groups was significant (Bonferroni-Holm corrected). **A.** Sex distribution for typical APs (left), other drugs (middle), and drug-naive (right) groups. **B.** Age distribution (in 5-year bins) for typical APs (left), other drugs (middle), and drug-naive (right) groups. **C.** Diagnosis (in ICD-10 codes) distribution for typical APs (left), other drugs (middle), and drug-naive (right) groups. **D.** Medicines taken count in typical APs (left), other drugs (middle), and drug-naive (right) groups. For this graph, distribution comparison statistics are calculated only for medicines other than typical APs.

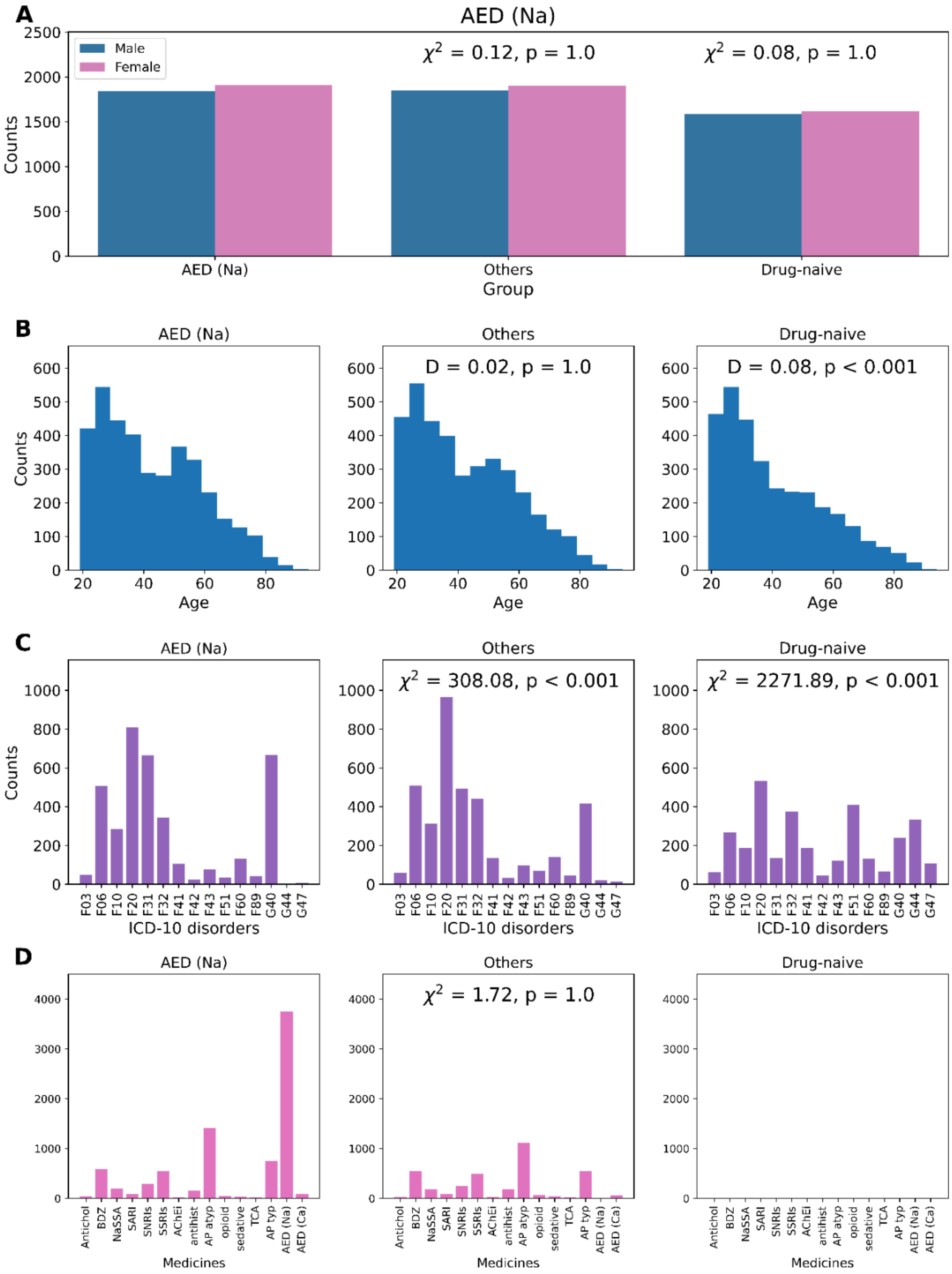

Figure S7. Age, sex, and diagnosis distributions for sodium-channel blocking anticonvulsant users and matched groups. Statistics (Kolmogorov–Smirnov D for age and X^2 for other graphs) and p-values show whether the difference between the distributions in the medicine group and other groups was significant (Bonferroni-Holm corrected). **A.** Sex distribution for anticonvulsants (left), other drugs (middle), and drug-naive (right) groups. **B.** Age distribution (in 5-year bins) for anticonvulsants (left), other drugs (middle), and drug-naive (right) groups. **C.** Diagnosis (in ICD-10 codes) distribution for anticonvulsants (left), other drugs (middle), and drug-naive (right) groups. **D**. Medicines taken count in anticonvulsants, other drugs (middle), and drug-naive (right) groups. For this graph, distribution comparison statistics are calculated only for medicines other than AED (Na).

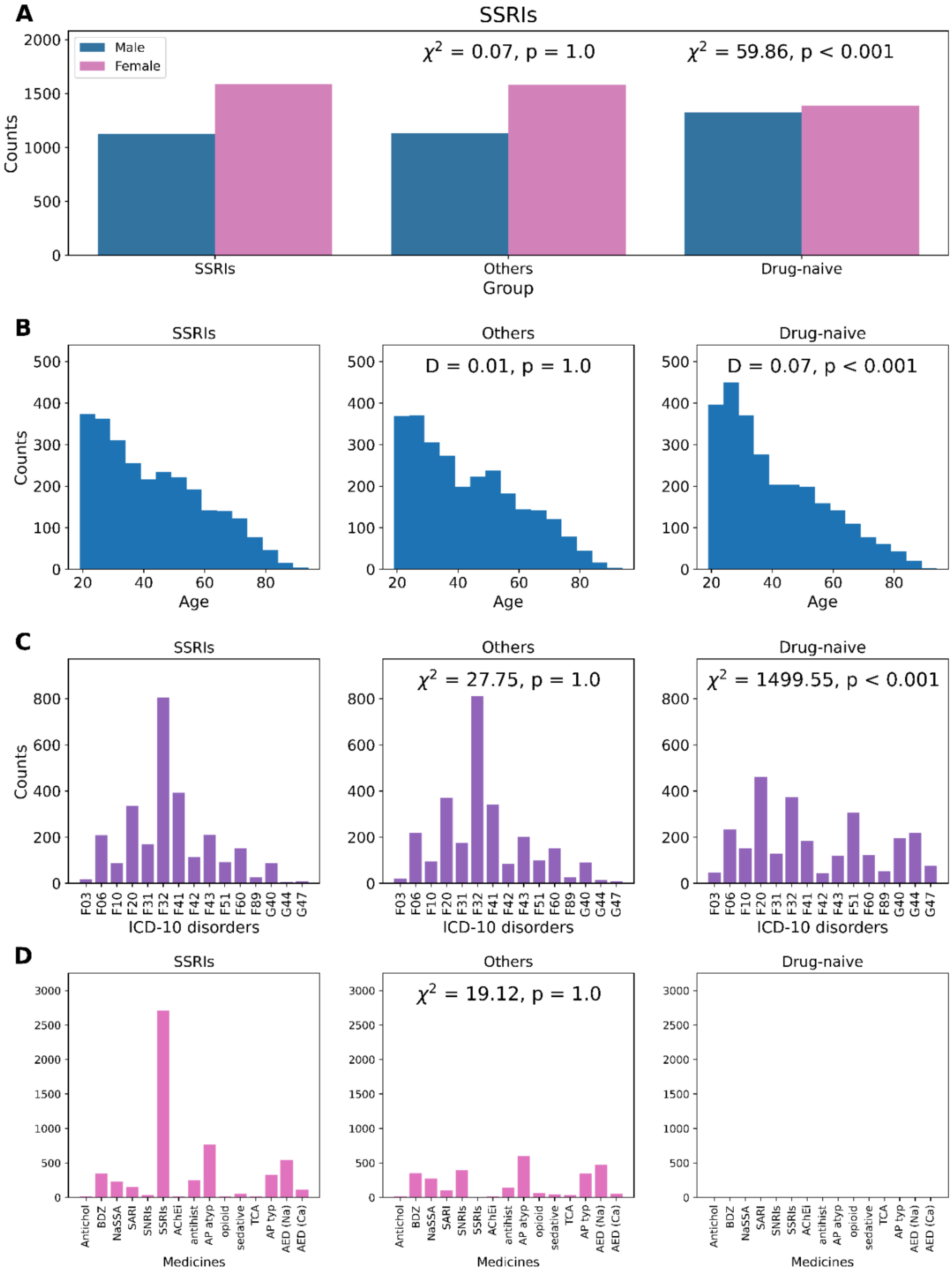

Figure S8. Age, sex, and diagnosis distributions for SSRIs users and matched groups. Statistics (Kolmogorov–Smirnov D for age and X^2 for other graphs) and p-values show whether the difference between the distributions in the medicine group and other groups was significant (Bonferroni-Holm corrected). **A.** Sex distribution for SSRIs (left), other drugs (middle), and drug-naive (right) groups. **B.** Age distribution (in 5-year bins) for SSRIs, other drugs (middle), and drug-naive (right) groups. **C.** Diagnosis (in ICD-10 codes) distribution for SSRIs, other drugs (middle), and drug-naive (right) groups. **D**. Medicines taken count in atypical SSRIs (left), other drugs (middle), and drug-naive (right) groups. For this graph, distribution comparison statistics are calculated only for medicines other than SSRI.

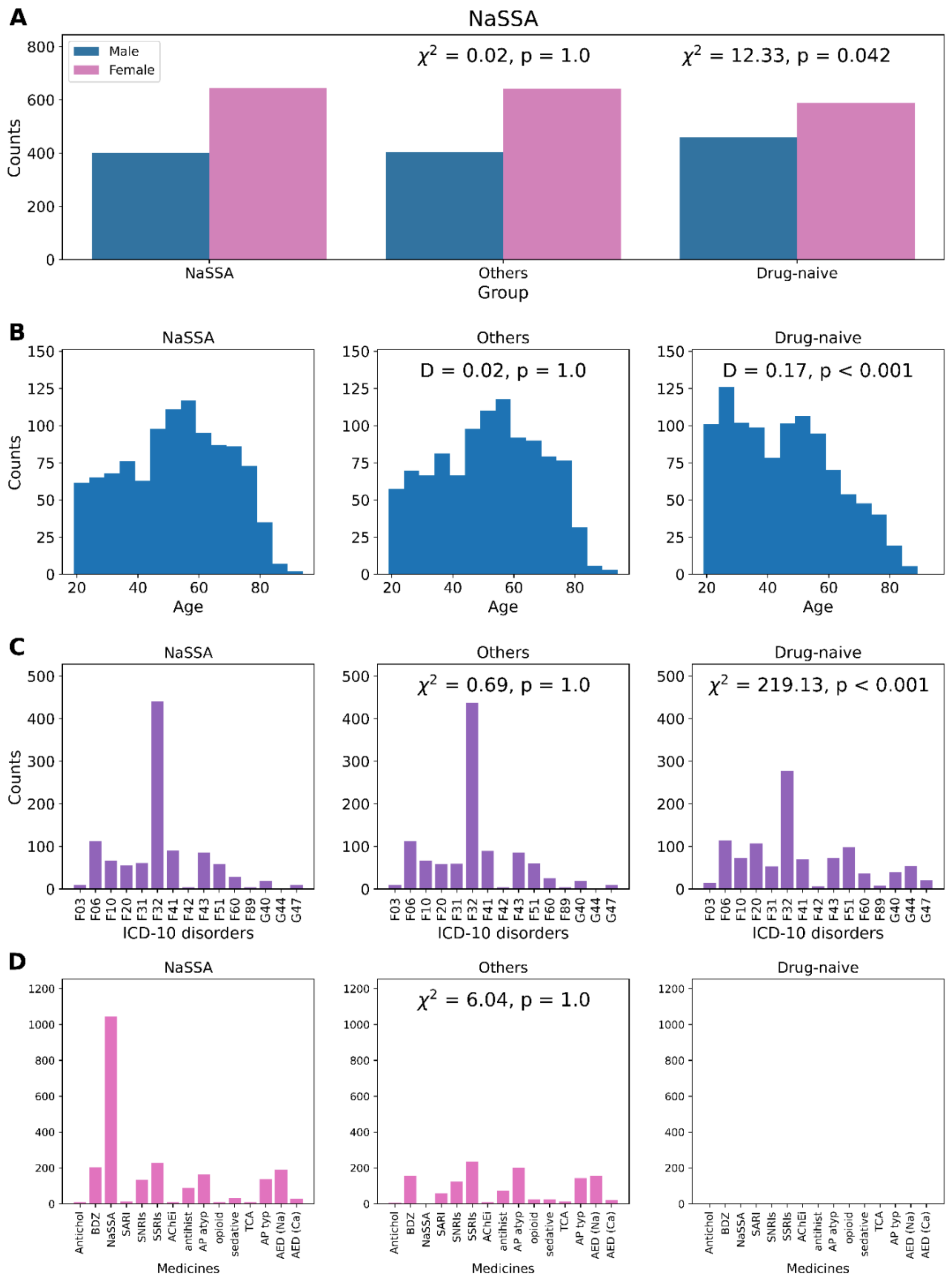

Figure S9. Age, sex, and diagnosis distributions for NaSSA users and matched groups. Statistics (Kolmogorov–Smirnov D for age and X^2 for other graphs) and p-values show whether the difference between the distributions in the medicine group and other groups was significant (Bonferroni-Holm corrected). **A.** Sex distribution for NaSSA (left), other drugs (middle), and drug-naive (right) groups. **B.** Age distribution (in 5-year bins) for NaSSA, other drugs (middle), and drug-naive (right) groups. **C.** Diagnosis (in ICD-10 codes) distribution for NaSSA, other drugs (middle), and drug-naive (right) groups. **D**. Medicines taken count in atypical NaSSA (left), other drugs (middle), and drug-naive (right) groups. For this graph, distribution comparison statistics are calculated only for medicines other than NaSSA.

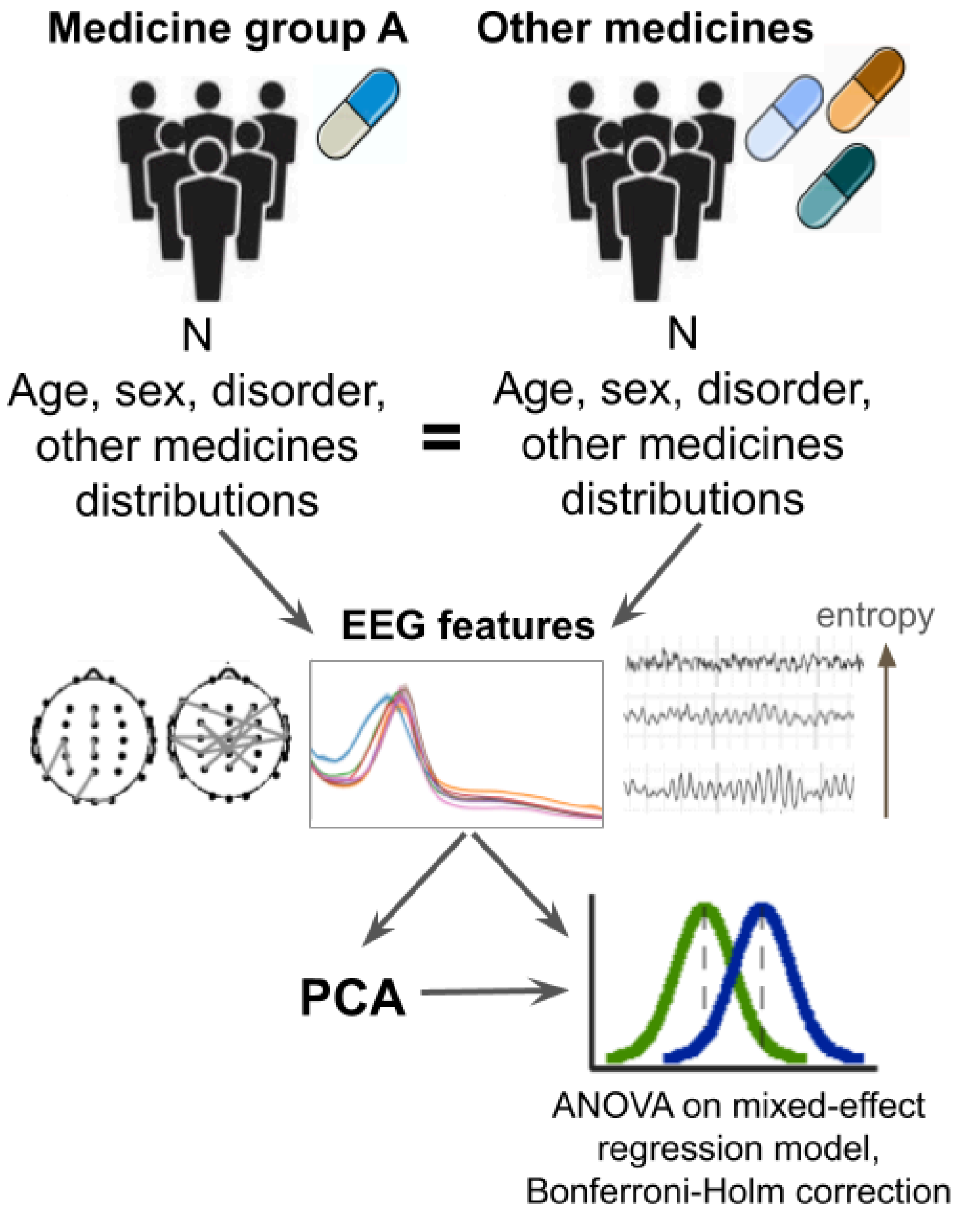

Figure S10. Graphical summary of the applied methodology.

Additional characteristics of the patients' sample

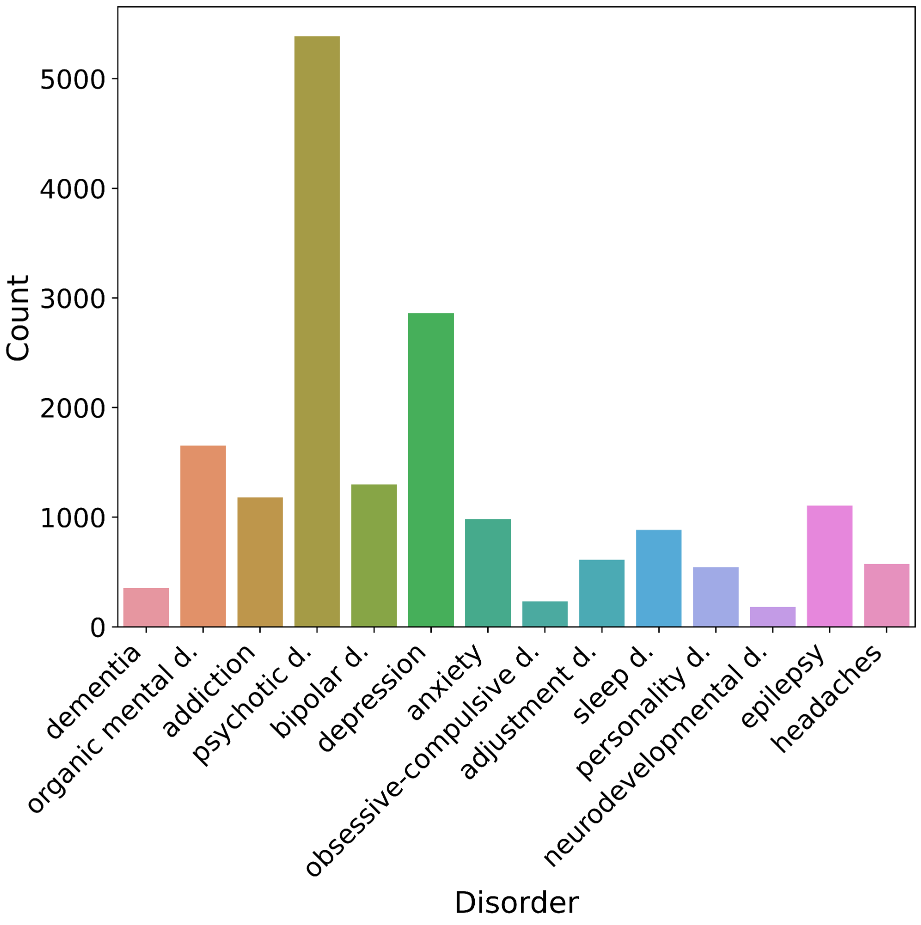

Figure S11. Disorder distribution among the patients.

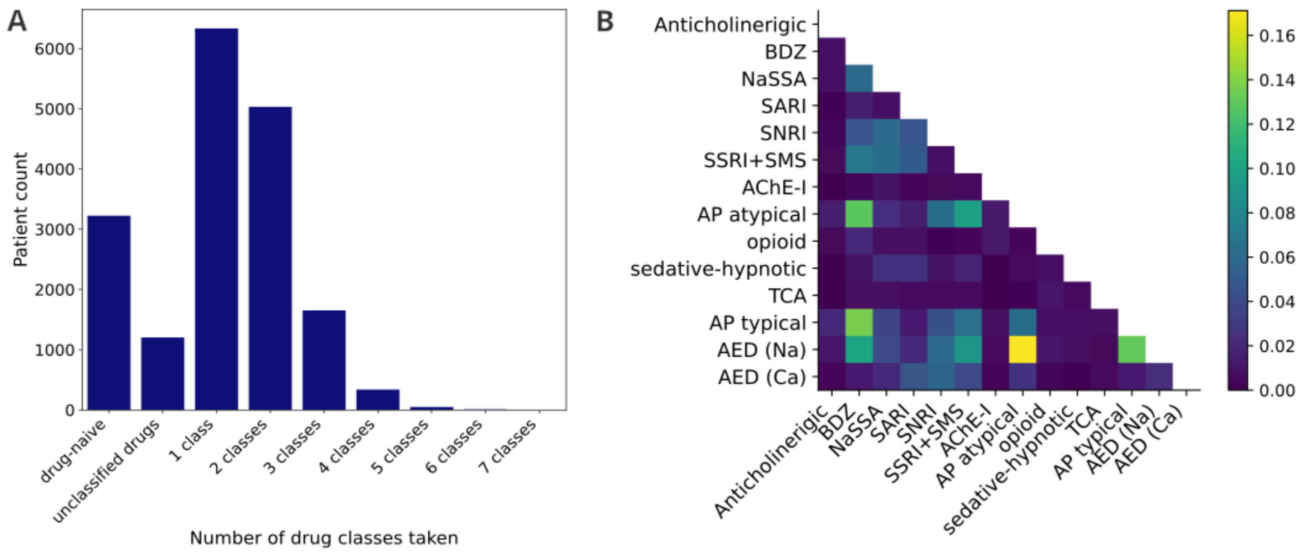

Figure S12. Multidrug therapy. A. Number of patients taking different numbers of drug classes. B. Jaccard similarity correlation matrix summarizing the most frequent multidrug therapy combinations.

Recording time and hospital site in comparison groups

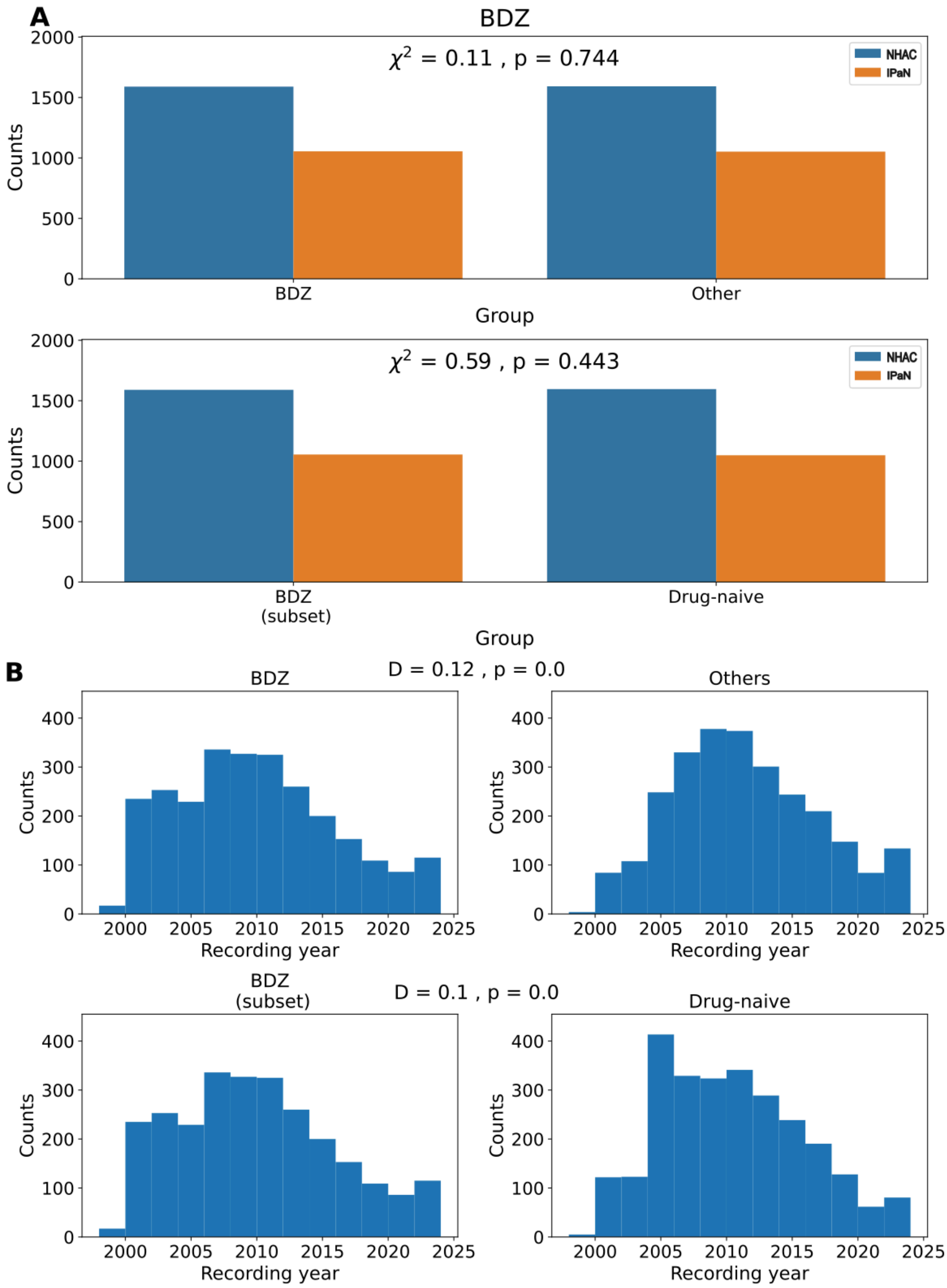

Figure S13. Hospital site and recording year distributions for BDZ users and matched groups. **A.** Hospital site for BDZ (top-left), other drugs (top-right), BDZ matched to drug-naive group (bottom-left), and drug-naive (bottom-right) groups. **B.** Date of the recording (in years) for BDZ (top-left), other drugs (top-right), BDZ matched to drug-naive group (bottom-left), and drug-naive (bottom-right) groups.

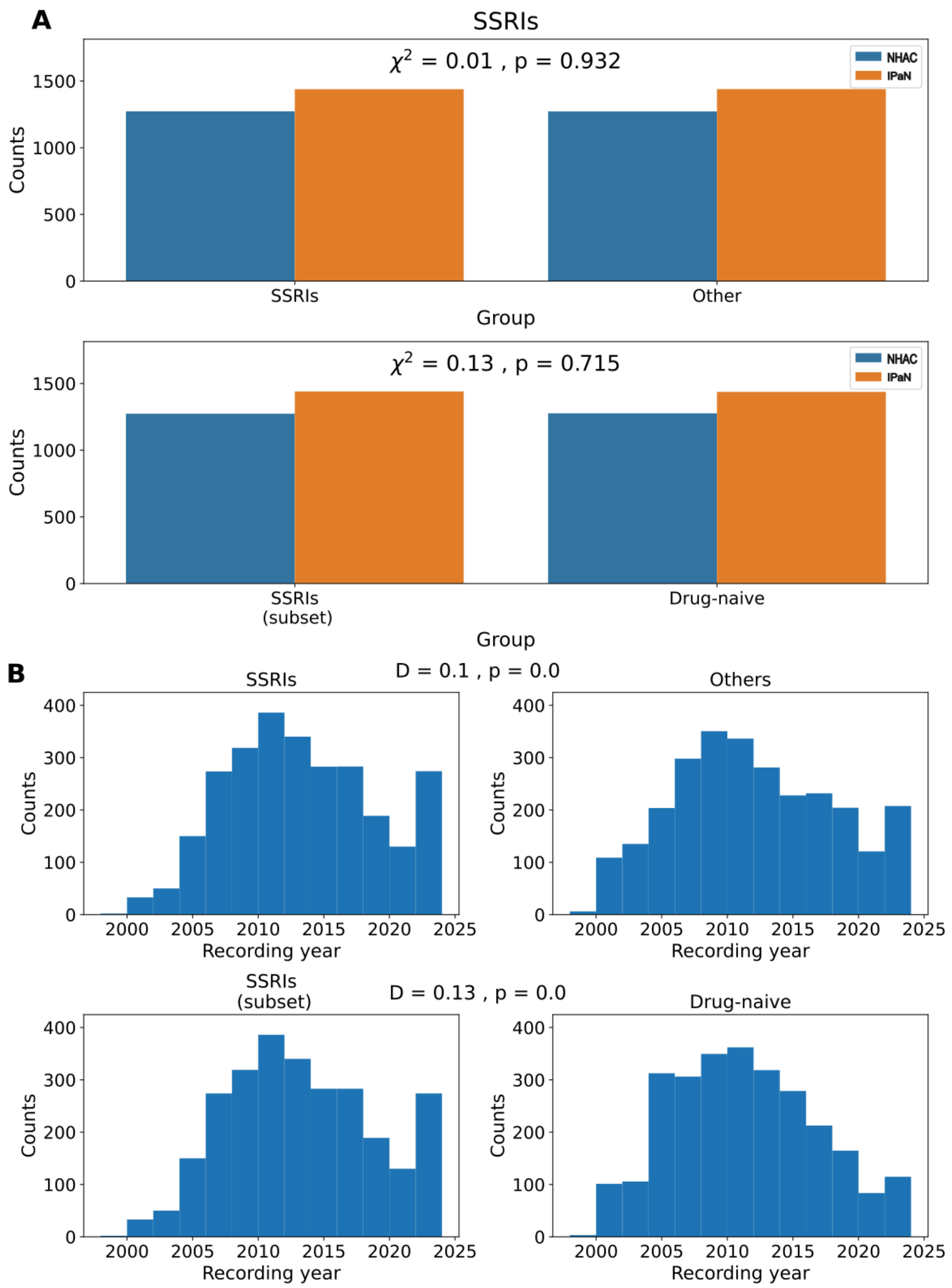

Figure S14. Hospital site and recording year distributions for SSRI users and matched groups. **A.** Hospital site for SSRI (top-left), other drugs (top-right), SSRI matched to drug-naive group (bottom-left), and drug-naive (bottom-right) groups. **B.** Date of the recording (in years) for SSRI (top-left), other drugs (top-right), SSRI matched to drug-naive group (bottom-left), and drug-naive (bottom-right) groups.

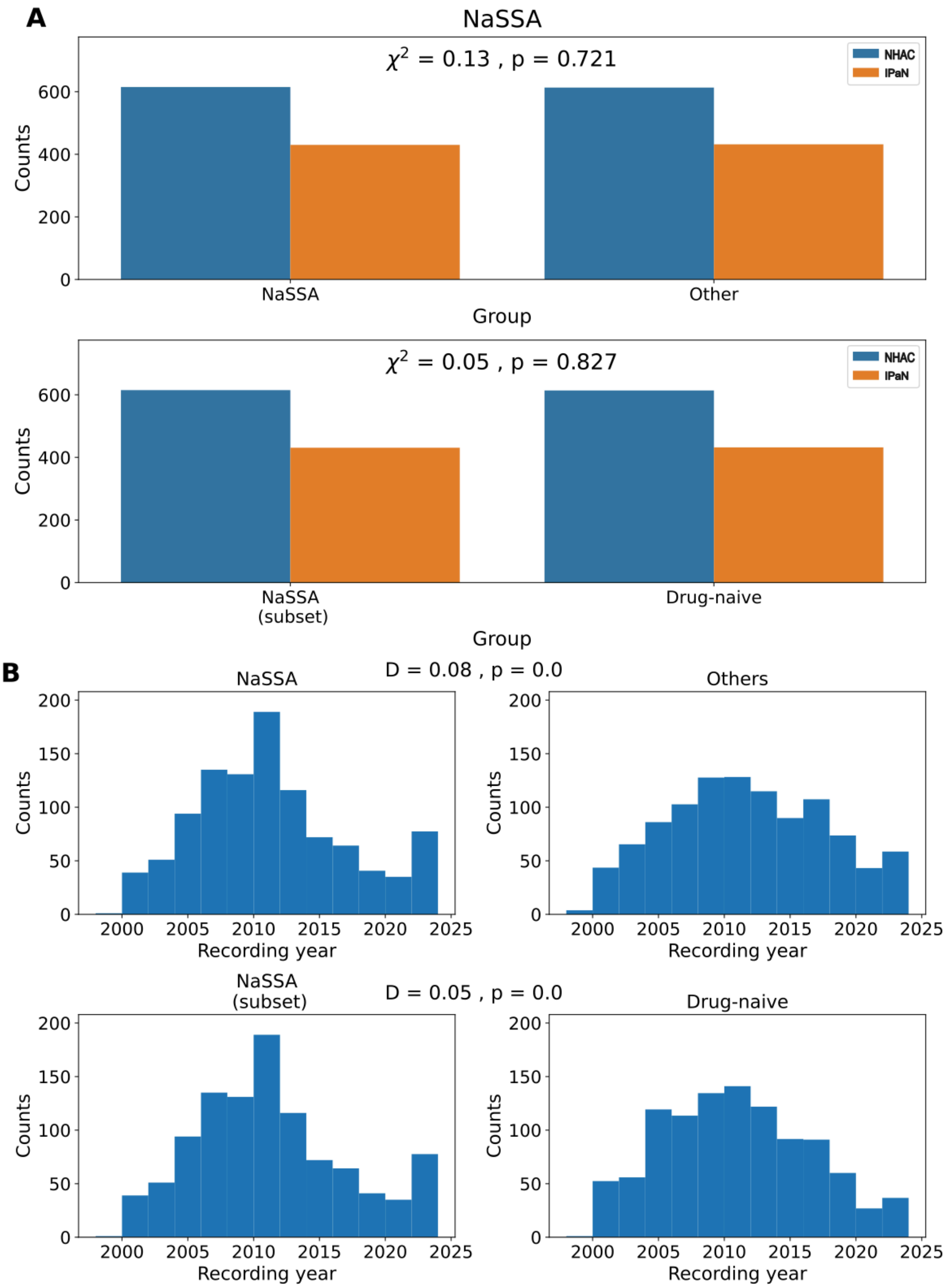

Figure S15. Hospital site and recording year distributions for NaSSA users and matched groups. **A.** Hospital site for NaSSA (top-left), other drugs (top-right), NaSSA matched to drug-naive group (bottom-left), and drug-naive (bottom-right) groups. **B.** Date of the recording (in years) for NaSSA (top-left), other drugs (top-right), NaSSA matched to drug-naive group (bottom-left), and drug-naive (bottom-right) groups.

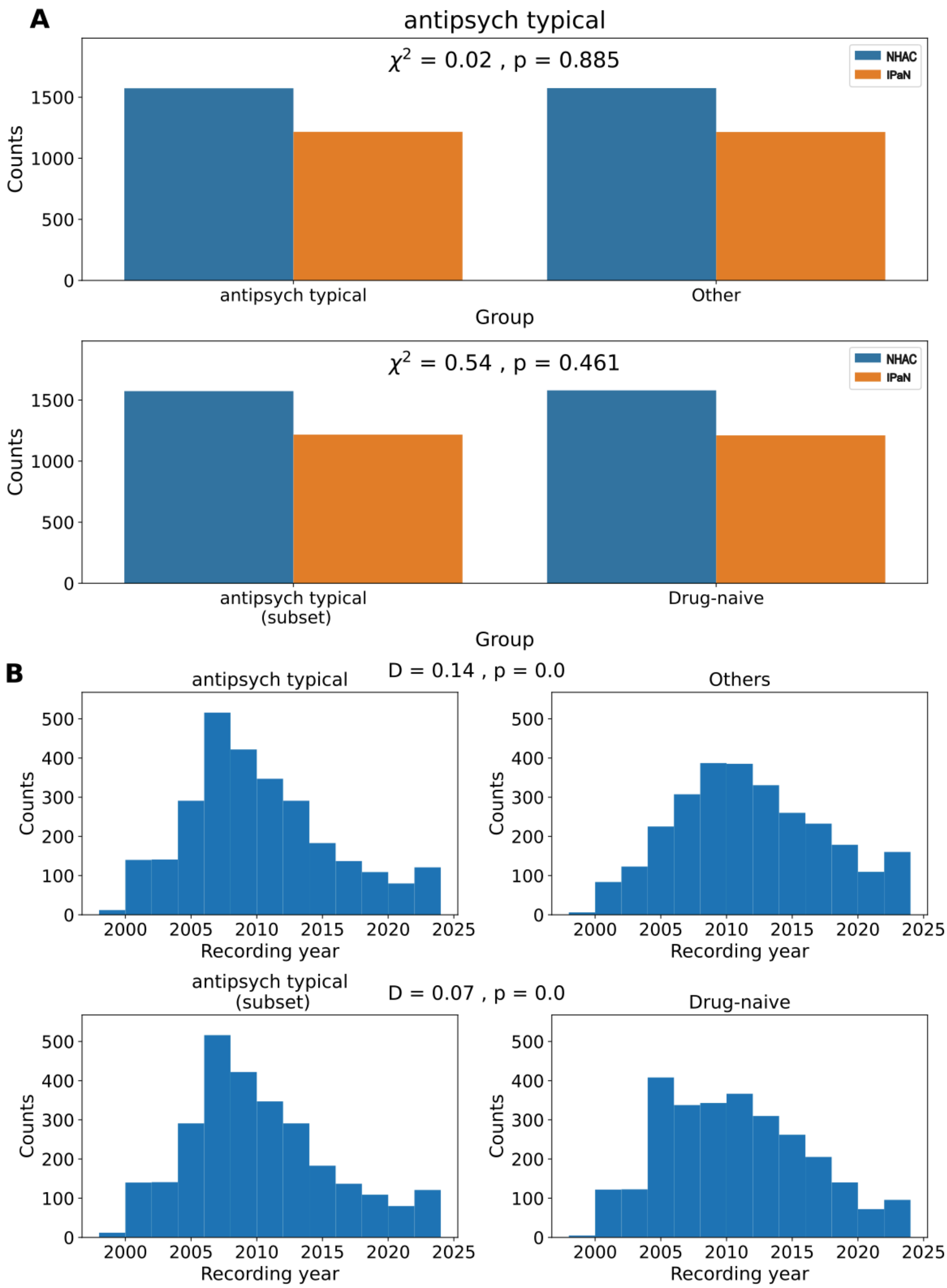

Figure S16. Hospital site and recording year distributions for typical AP users and matched groups. **A.** Hospital site for AP typical (top-left), other drugs (top-right), AP typical matched to drug-naive group (bottom-left), and drug-naive (bottom-right) groups. **B.** Date of the recording (in years) for AP typical (top-left), other drugs (top-right), AP typical matched to drug-naive group (bottom-left), and drug-naive (bottom-right) groups.

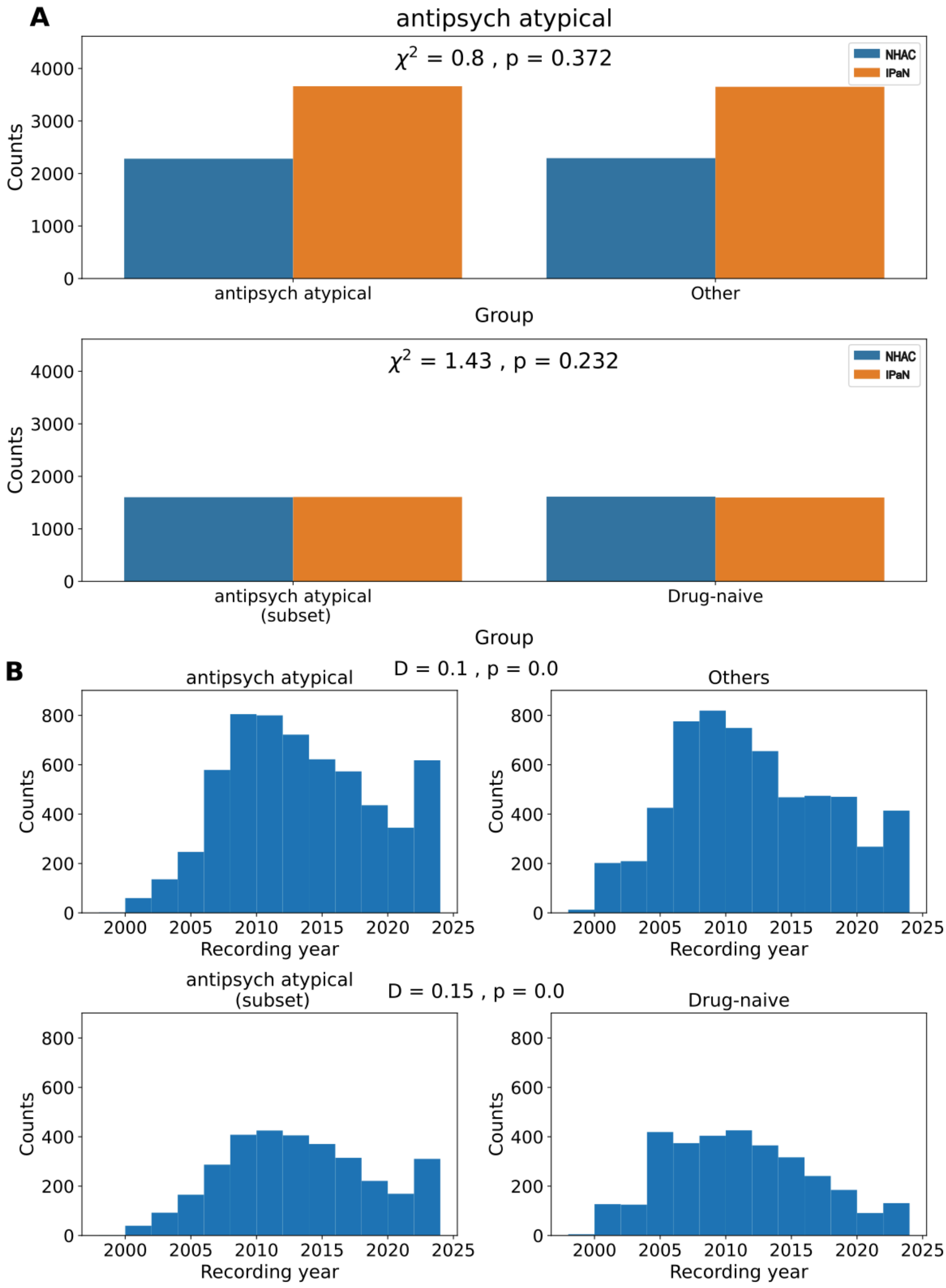

Figure S17. Hospital site and recording year distributions for atypical AP users and matched groups. **A.** Hospital site for AP atypical (top-left), other drugs (top-right), AP atypical matched to drug-naive group (bottom-left), and drug-naive (bottom-right) groups. **B.** Date of the recording (in years) for AP atypical (top-left), other drugs (top-right), AP atypical matched to drug-naive group (bottom-left), and drug-naive (bottom-right) groups.

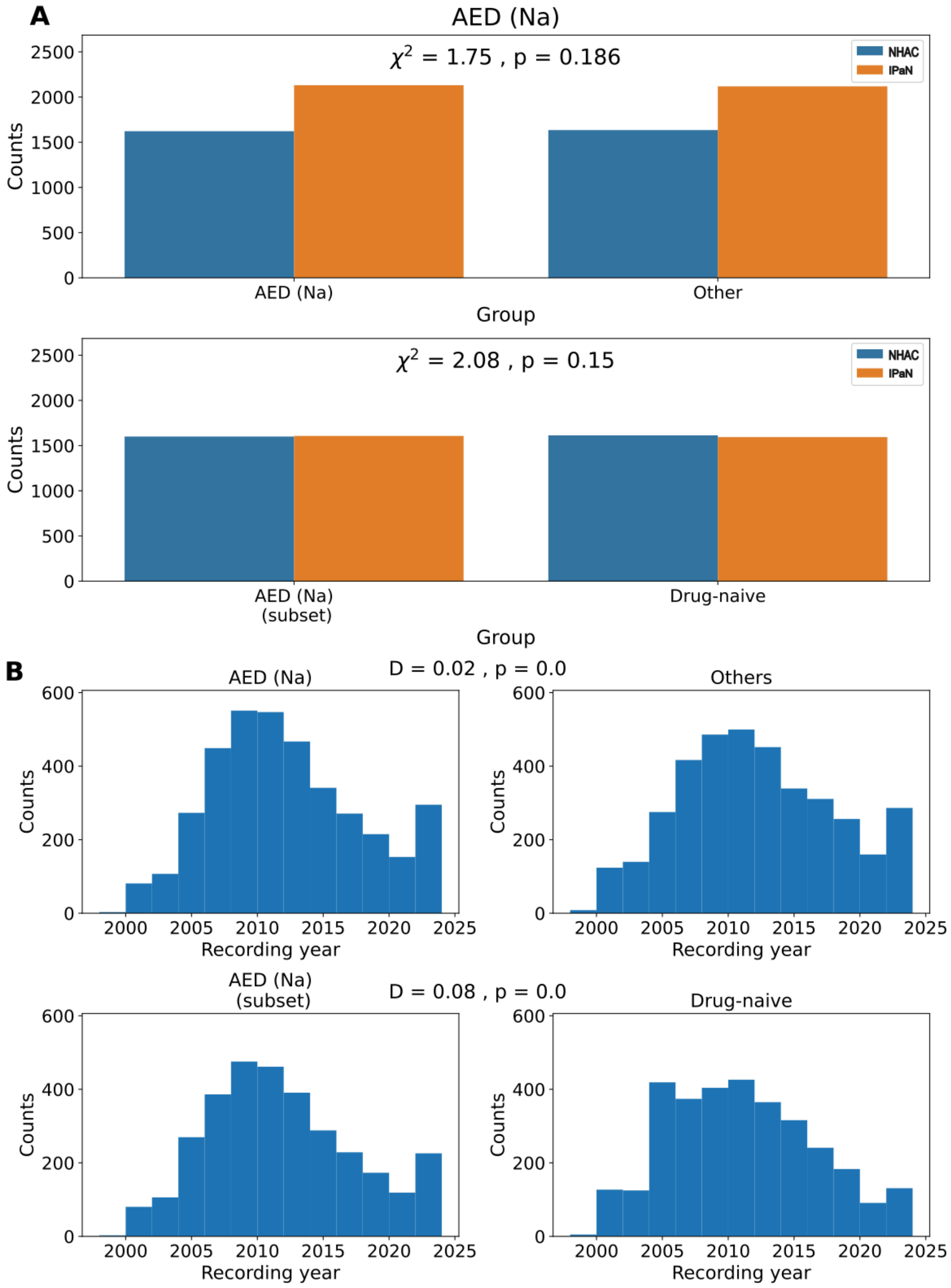

Figure S18. Hospital site and recording year distributions for sodium-channel blocking anticonvulsant (AED Na) users and matched groups. **A.** Hospital site for AED Na (top-left), other drugs (top-right), AED Na matched to drug-naive group (bottom-left), and drug-naive (bottom-right) groups. **B.** Date of the recording (in years) for AED Na (top-left), other drugs (top-right), AED Na matched to drug-naive group (bottom-left), and drug-naive (bottom-right) groups.

Preprocessing

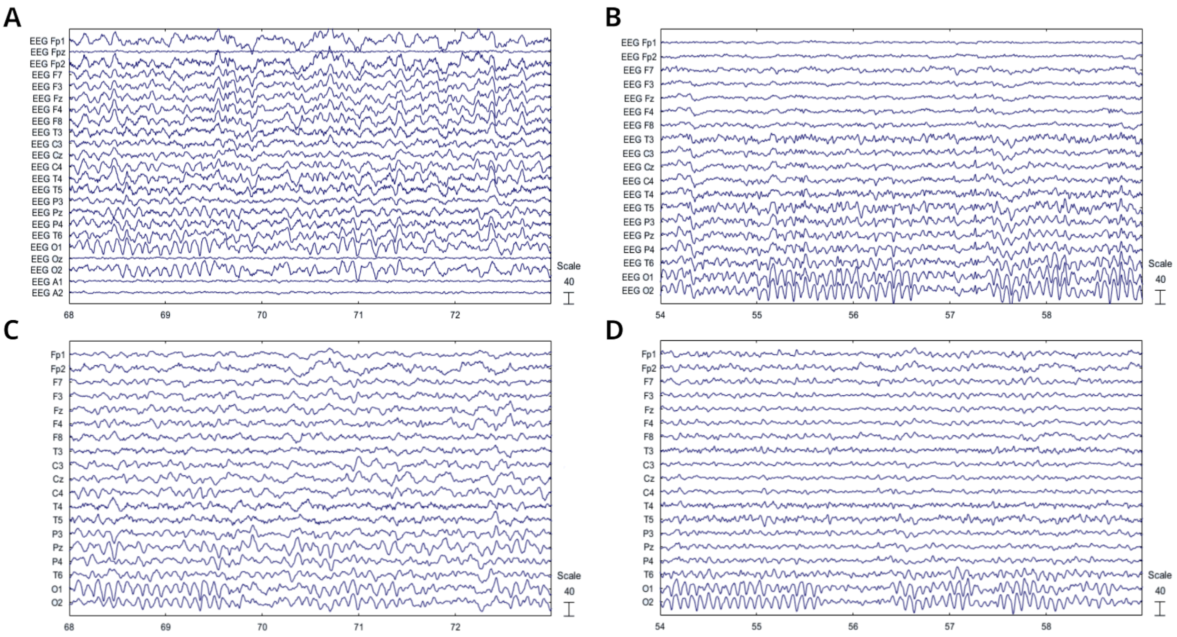

Figure S19. Representative recordings from the two hospitals, before (top) and after (bottom) preprocessing and data unification. **A.** Representative EEG recording fragment from NHAC hospital. **B.** Representative file from IPaN hospital. **C, D.** The same fragments after rereferencing, resampling, and artifact reduction.

Table S7. Preprocessing pipeline pseudocode

1. Read .edf EEG file (and events)
#Channels<br>2. Standardize channel names<br>3. Pick 19 channels present in every file<br>4. Add channel localization from a standard 10-20 montage file<br>5. Interpolate bad channels (spherical spline interpolation, threshold: kurtosis > 6 SD)
#Basic preprocessing and rereferencing<br>6. Clean line noise (EEGlab Cleanline plugin)<br>7. Re-reference to average and then REST rereference<br>8. Filtering - 1.5 highpass
#Artifact cleaning<br>9. Clean raw data with ASR with high threshold (window criterion: 0.55 channels > 20 SD) - removes highly noisy fragments of data<br>10. Check if events were removed by ASR and insert the removed events in the closest retained sample<br>11. Interpolate bad channels (>2 out of 4 signal properties > 3 SD) within 1-s fragments (FASTER eeglab plugin)
12. If length(removed_fragments) < length(EEG-raw)/2<br>save file<br>else<br>file considered bad and rejected

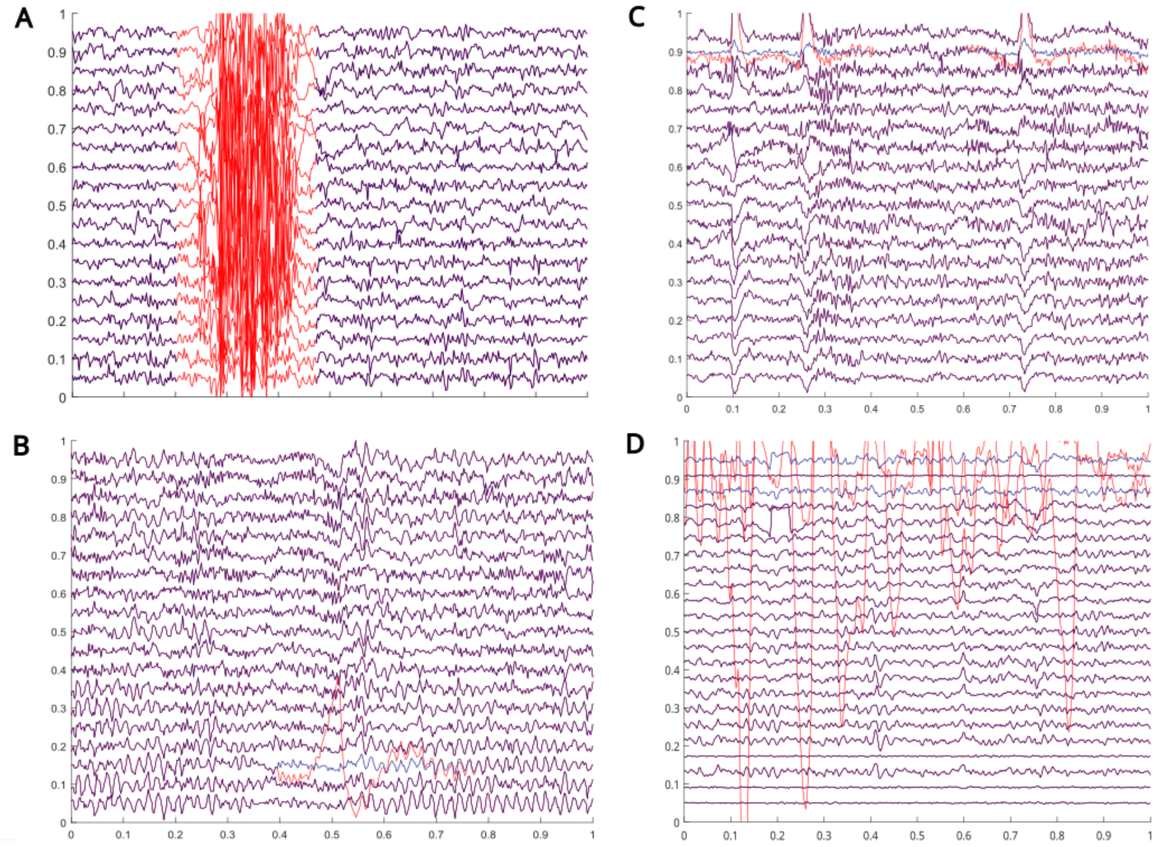

Figure S20. Examples of artifact handling by our preprocessing algorithm. Data before preprocessing are plotted in red, while data after preprocessing are plotted in blue. Purple color indicates the data has not changed. **A.** Muscle artifact. The red section was cut off. **B.** Electrode losing contact artifact interpolated. **C.** Eye blinks interpolated. **D.** Bad channels interpolated.
